# Ploidy evolution in a wild yeast is linked to an interaction between cell type and metabolism

**DOI:** 10.1371/journal.pbio.3001909

**Published:** 2023-11-09

**Authors:** Johnathan G. Crandall, Kaitlin J. Fisher, Trey K. Sato, Chris Todd Hittinger

**Affiliations:** 1 Laboratory of Genetics, Wisconsin Energy Institute, J. F. Crow Institute for the Study of Evolution, Center for Genomic Science Innovation, University of Wisconsin-Madison, Madison, Wisconsin, United States of America; 2 DOE Great Lakes Bioenergy Research Center, University of Wisconsin-Madison, Madison, Wisconsin, United States of America; Stowers Institute for Medical Research, UNITED STATES

## Abstract

Ploidy is an evolutionarily labile trait, and its variation across the tree of life has profound impacts on evolutionary trajectories and life histories. The immediate consequences and molecular causes of ploidy variation on organismal fitness are frequently less clear, although extreme mating type skews in some fungi hint at links between cell type and adaptive traits. Here, we report an unusual recurrent ploidy reduction in replicate populations of the budding yeast *Saccharomyces eubayanus* experimentally evolved for improvement of a key metabolic trait, the ability to use maltose as a carbon source. We find that haploids have a substantial, but conditional, fitness advantage in the absence of other genetic variation. Using engineered genotypes that decouple the effects of ploidy and cell type, we show that increased fitness is primarily due to the distinct transcriptional program deployed by haploid-like cell types, with a significant but smaller contribution from absolute ploidy. The link between cell-type specification and the carbon metabolism adaptation can be traced to the noncanonical regulation of a maltose transporter by a haploid-specific gene. This study provides novel mechanistic insight into the molecular basis of an environment–cell type fitness interaction and illustrates how selection on traits unexpectedly linked to ploidy states or cell types can drive karyotypic evolution in fungi.

## Abstract 2

Ploidy is a fundamental trait of all organisms, but it varies within and between fungal species. This study reveals how strong conditional selection on a metabolic trait can lead to ploidy and cell type evolution in the wild yeast Saccharomyces eubayanus.

## Introduction

Ploidy is a fundamental aspect of the biology of all organisms, but it is subject to striking diversity across the tree of life—between related species, between individuals of the same species, and within individuals across cell types and life cycles [1]. The long-term impact of ploidy variation on eukaryotic evolution, particularly as a mechanism for generating raw material for natural selection, has long been recognized [2–6]. Recent work, primarily in the model eukaryote *Saccharomyces cerevisiae*, has further defined short-term evolutionary consequences of different ploidy states [7–13]. It remains less clear, however, what immediate effects on organismal fitness a ploidy transition can engender. In *S*. *cerevisiae*, ploidy variation is present both within the natural life cycle [14] and among isolates from diverse environments [15,16]. Despite this natural variation, diploidy seems to be generally favored [15]. Indeed, diploids frequently arise and sweep to fixation in laboratory evolution experiments founded with non-diploid strains [7,17–22].

In the limited cases where a direct fitness advantage of diploidy has been found in *S*. *cerevisiae* in the absence of confounding variation, the specific molecular bases have remained elusive. A large-scale survey of *S*. *cerevisiae* and its sister species *Saccharomyces paradoxus* suggested that specific ploidy-by-environment interactions were necessary to explain observed differences in fitness proxies between haploids and diploids, which argues against generalizable predictions of fitness effects of ploidies across environments [23]. Similar experiments in *Candida albicans* found genetic background to influence fitness more than ploidy in several conditions that might be predicted to favor different ploidy states [24]. By contrast, more recent work capturing a wide swath of genetic diversity in *Saccharomyces eubayanus*, which diverged from *S*. *cerevisiae* approximately 17 million years ago [25], failed to find meaningful differences in phenotypic traits between ploidies [26].

Adding complexity to the interpretation and prediction of fitness differences between ploidy states in yeasts is the nuanced relationship between ploidy and cell type across species. In wild-type *Saccharomyces*, for example, ploidy indirectly controls cell type through the presence or absence of alleles at a single locus, the *MAT*ing type locus [14,27,28]. The haploid cell types express a common set of haploid-specific genes, as well as mating type-specific genes dependent on the allele present at the *MAT* locus, while diploids repress these gene sets but are competent to induce the expression of a small number of genes under specific conditions (e.g., meiosis). Although investigations into ploidy-specific fitness effects have primarily focused on the physiological differences between haploids and diploids that are independent of cell type, it remains plausible that underappreciated aspects of cell-type specification could influence traits that in turn impact organismal fitness.

Perhaps, the most compelling evidence for widespread effects of selection on cell type across fungi can be found among pathogenic species. Highly skewed mating type ratios have been described among isolates of *Cryptococcus neoformans*, *Candida glabrata*, *Candida auris*, *Fusarium poae*, and *Fusarium verticillioides* [29–35]. Large mating type skews are also found in clinical isolates of *Aspergillus fumigatus* but not in isolates from other sources, and mating type has been shown to influence pathogenicity in vitro and virulence in vivo in this species [36,37]. Similar links between mating type and virulence traits have been suggested in *Cr*. *neoformans*, *C*. *auris*, *Mucor iregularis*, and *Fusarium graminearum* [38–46], suggesting that unexpected links between cell type and traits experiencing intense selection may be widespread among fungi.

Microbial traits and their underlying genotypes are of particular interest when they directly impact human health, are important for biotechnological processes, or serve as models of eukaryotic evolution. The latter 2 cases are exemplified in the emerging model yeast *S*. *eubayanus*, the wild parent of hybrid lager-brewing yeast. Since its isolation as a pure species [47], *S*. *eubayanus* has become a model for microbial population genomics and ecology [48–53], as well as a key target for applied biotechnological research [54–59]. A focal ecological and industrial trait in this wild species is the ability to consume and metabolize the α-glucoside maltose, which is the most abundant sugar in the wort used to brew beer [60,61]. This trait is nearly ubiquitous among isolates of *S*. *eubayanus* and its sister species *Saccharomyces uvarum* [62], but it has been lost [63] or severely curtailed [48,49,64,65] in the Holarctic subpopulation of *S*. *eubayanus*, a low-genetic diversity lineage broadly distributed across the northern hemisphere that contains the closest identified relatives to the *S*. *eubayanus* subgenome of hybrid lager-brewing yeasts [51,53]. Paradoxically, the genomes of Holarctic *S*. *eubayanus* strains contain functional structural maltose metabolism genes, which appear to be inefficiently expressed in the presence of maltose [63,64]. Because the *cis*-regulatory logic of at least some of these structural genes appears to have been retained, it has been proposed that the *trans*-regulating proteins may have been rendered nonfunctional [63], at least with regards to their homology-predicted activities.

In an effort to identify mechanisms by which maltose utilization might be refined or regained after secondary loss, we previously subjected a wild diploid *S*. *eubayanus* strain from the Holarctic subpopulation to adaptive laboratory evolution (ALE) under selection for improved growth on maltose [64]. Here, we map the genetic basis of adaptation in the evolved clones. We find that, surprisingly, haploids emerged and rose to high frequency in replicate ALE populations founded with this diploid strain, which is a highly unusual ploidy transition for *Saccharomyces*. We find that haploidy confers a substantial fitness advantage in the ALE conditions, but that haploids experience a fitness tradeoff in rich conditions, consistent with previous observations of diploid advantage in *S*. *cerevisiae*. We identify cell type as the primary driver of adaptive fitness, with a smaller but significant contribution from absolute ploidy. Finally, we demonstrate that a major fitness-modifying gene has elevated expression in evolved haploids, and that this effect is linked to unexpected regulation by a haploid-specific transcription factor that regulates invasive growth in *S*. *cerevisiae*. Our results suggest a mechanism underlying a ploidy-by-environment fitness effect and demonstrate how strong selection on traits linked to cell types can drive karyotypic evolution in fungi.

## Results and discussion

### Evolved *S*. *eubayanus* isolates harbor mutations incongruous with ancestral ploidy

**Fig 1:**
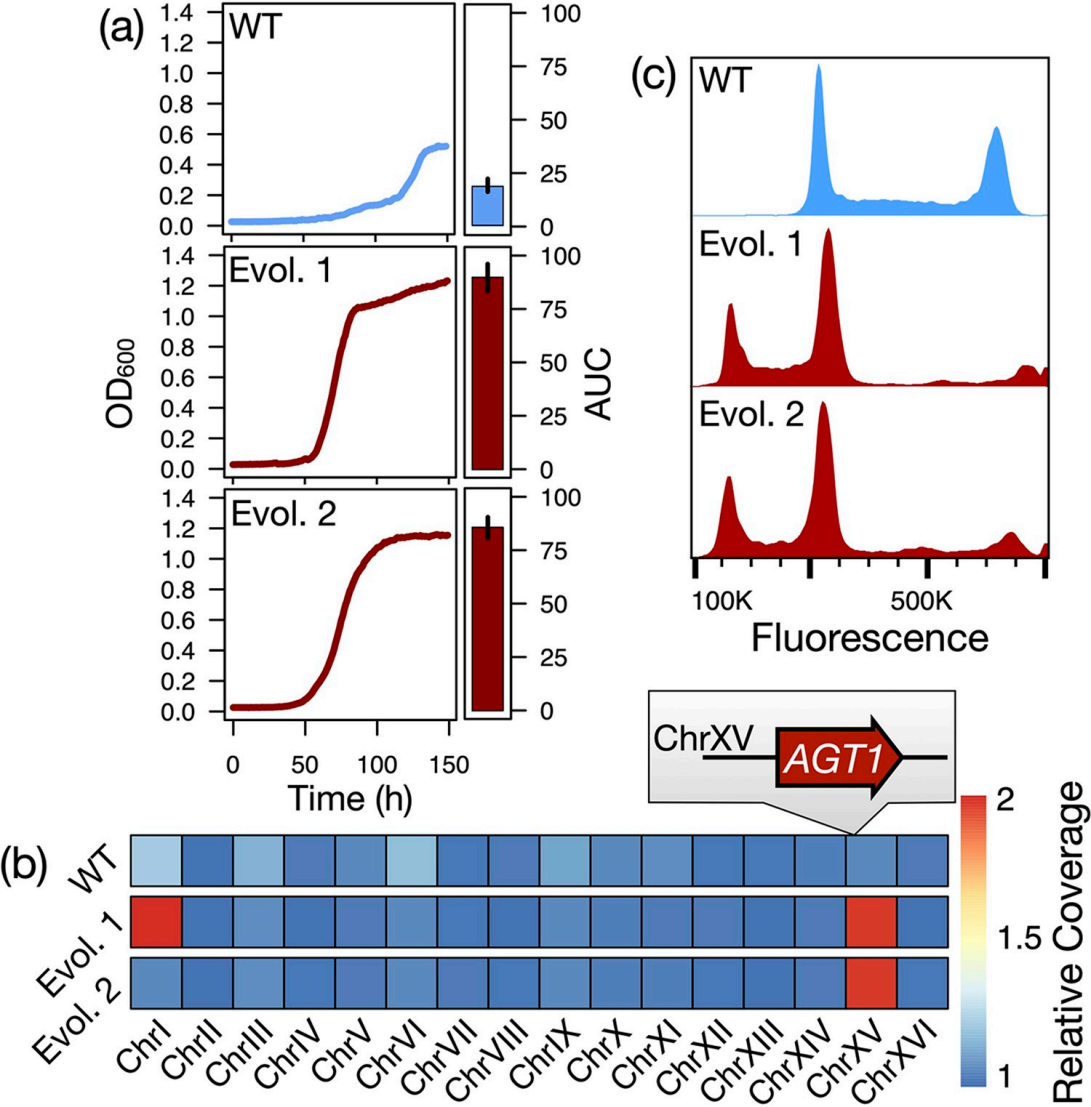
Phenotypic and karyotypic evolution of *S*. *eubayanus* isolates. (a) Growth of the wild *S*. *eubayanus* strain (WT) and clonal isolates from 2 replicate experimental evolution populations (Evol. 1, Evol. 2) on maltose. A representative growth curve is shown for each; bar plots show mean and standard error of total growth (AUC) across 6 biological replicates for each genotype. (b) Relative copy number of each chromosome in the WT and evolved strains, inferred from sequencing depth. The parallel ChrXV gain includes a homolog of an *S*. *cerevisiae* gene encoding an α-glucoside transporter (*AGT1*/YGR289C). (c) Smoothed histograms of cellular DNA content in the WT and evolved strains as measured by flow cytometry. Fluorescence intensity is proportional to DNA content; primary peaks correspond to cells in G1 and G2. The data underlying this figure can be found in S1 Data. AUC, area under the curve; WT, wild-type.

We previously experimentally evolved a wild strain of *S*. *eubayanus* from the Holarctic subpopulation under selection for improved growth on the industrially relevant α-glucoside maltose [64]. We picked clones from 2 replicate populations of the ALE experiment that displayed significantly increased growth (*p* = 0.002, Mann–Whitney *U* tests) on maltose compared to the ancestral strain (Fig 1A). To map the genetic basis of improved growth on maltose, we sequenced the genomes of each clone to a final average depth of 95-fold. We mapped these reads to a re-sequenced and annotated assembly of the ancestral strain and identified a total of 4 single-nucleotide polymorphisms (SNPs) and 3 large-scale copy number variants (CNVs) in the form of aneuploidies across the evolved isolates (Fig 1B and S3 Table). We did not identify single-nucleotide variants in or near any genes with clear relationships to α-glucoside metabolism, although 1 SNP introduced a premature stop codon in *IRA1*, a common target of adaptive mutations in batch-style experimental evolution [7,22,66–68]. One aneuploidy (ChrXV gain) was shared between evolved isolates and encompassed a homolog of the *S*. *cerevisiae* generalist α-glucoside transporter *AGT1*/YGR289C, suggesting a potential mechanism for adaptation (Fig 1B); we did not detect further copy number expansion of this gene in the evolved isolates (S1 Fig).

Unexpectedly, all SNPs in the evolved isolates were represented by a single, non-reference allele (S2 Fig). Although mitotic recombination can generate losses of heterozygosity at new or standing variation during adaptive evolution [69–75], our results differed significantly from 2 recent large-scale experimental evolution studies in *S*. *cerevisiae*, which found approximately 5% to 10% of mutations to be homozygous in diploid or autodiploid clones after 4,000 generations [7,9]. In comparison, our observed allele frequencies at mutated sites are highly improbable under the null expectation of diploidy (binomial tests: *p* = 5.3 × 10^−6^, *p* = 1 × 10^−4^, respectively). Thus, we reasoned that the observed patterns in allele frequency might best be explained by an unexpected and atypical ploidy reduction to haploidy during ALE.

### Haploids emerged and rose to high frequency in diploid-founded populations

We directly determined the ploidy states of the evolved clones and the ancestral strain using flow cytometry (Fig 1C) and confirmed that the strain that was used to found the experimental populations was diploid (S3A Fig). Consistent with the results of genome sequencing, we found that clones from both ALE replicates had become haploid (Fig 1C). To test whether the clonal isolates we analyzed were simply from a rare and nonrepresentative subpopulation, we assayed the ploidy states present at the population level in both replicates of the ALE experiment (S3B Fig). Haploids were clearly detectable in each replicate by approximately 100 and 250 generations, respectively. As an orthogonal approach, we plated cells from the terminal time point of each population of the ALE experiment and used a PCR assay to genotype the *MAT* locus of single colonies. By this method, haploids constituted 74% to 100% of the cells we genotyped in the 2 ALE populations (S3C Fig). All haploids genotyped by PCR were found to be *MAT***a**, as were both sequenced isolates. Thus, although haploids may not have swept to fixation in both experimental populations, they repeatedly emerged and rose to high frequency over the duration of the ALE experiment.

### Haploids exhibit a direct condition-dependent fitness advantage

The abundance of haploids in our experimental populations could be explained by 2 alternative models: haploids might have a direct fitness advantage, or they might benefit indirectly from increased adaptability in our ALE environment. Two well-documented lines of evidence from previous studies seemed to strongly favor the latter hypothesis. First, *S*. *cerevisiae* haploids have repeatedly been shown to adapt more rapidly than diploids during experimental evolution, in part due to dominance effects at adaptive targets and ploidy-specific mutation rates and spectra; even large-scale mutations, such as aneuploidies, can have different fitness effects in different ploidies [7–12,76,77]. Second, *S*. *cerevisiae* displays a strong trend of converging on a diploid state during experimental evolution initiated with non-diploid strains [18]. Although theory predicts that haploids may be better able to meet their metabolic needs in nutrient-limiting conditions due to increased cell surface area-to-volume ratios, experimental evidence in yeast has failed to find widespread support for such generalizable trends [23,78–81], and our experimental evolution conditions could not strictly be considered to be limited in key nutrients. Given the relative simplicity of testing for differences in fitness between ploidies, we first sought to support or refute the model of direct haploid advantage.

**Fig 2:**
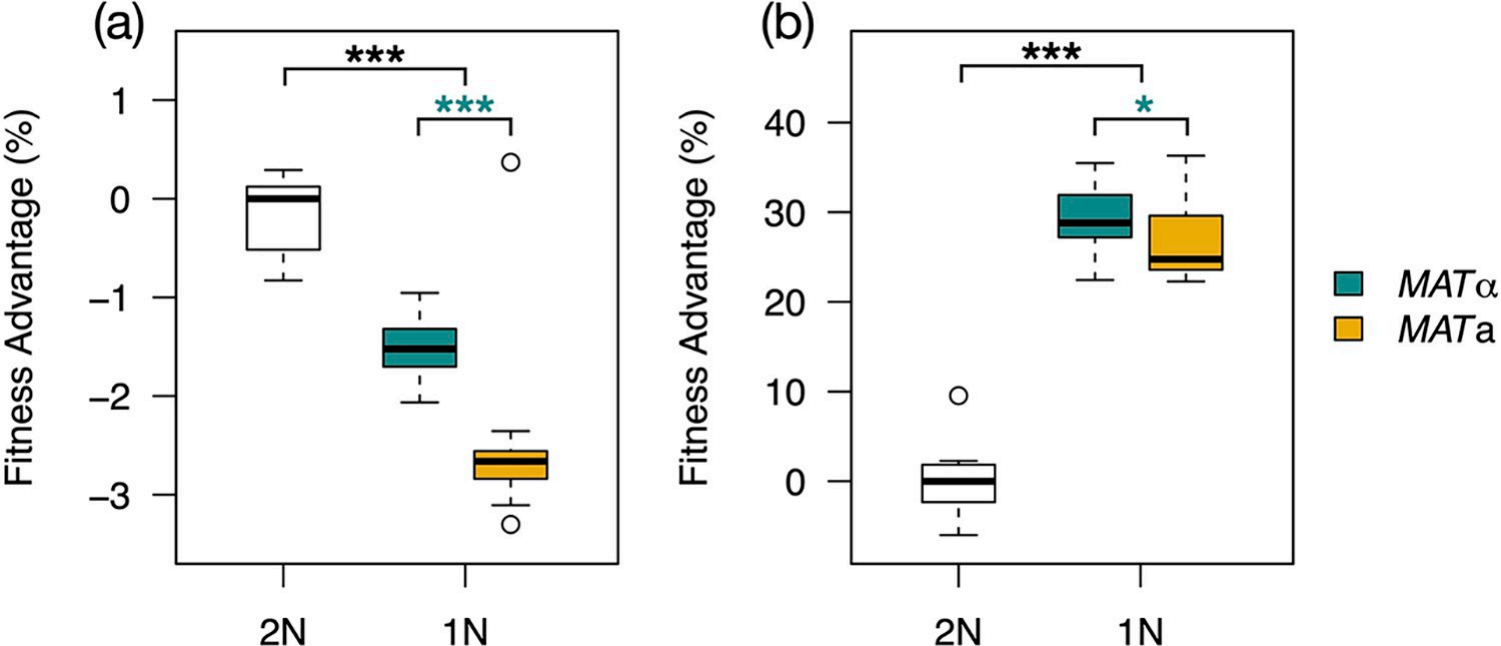
Haploids have a conditional fitness advantage. Boxplots show fitness measurements of isogenic diploids (*n* = 12) and haploids from fully viable tetrads (*n* = 47) in rich medium (a) and ALE conditions (b). *** *p* < 10^−4^ (Mann–Whitney *U* tests) between diploids and each haploid group (black) or between haploid groups (teal). In ALE conditions, the significance level between haploid groups was 0.013 (*). A diploid outlier in (a) at −6.6% is truncated from the plot space. The data underlying this figure can be found in S1 Data. ALE, adaptive laboratory evolution.

We used a sensitive competition assay to measure the fitness of isogenic diploids and haploids in the wild-type strain background following *HO* deletion, sporulation, and tetrad dissection. Consistent with observations in *S*. *cerevisiae* of direct or cryptic diploid advantage [7,15,17,18,20,22,82], haploids in our strain background exhibited median fitness defects of 1.5% (*p* = 1.3 × 10^−5^) to 2.7% (*p* = 9.9 × 10^−5^, Mann–Whitney *U* tests) relative to the isogenic diploid in rich medium (Fig 2A). By contrast, in the ALE conditions, haploids displayed median fitness advantages of 24.8% (*p* = 1.6 × 10^−9^) to 28.8% (*p* = 2.4 × 10^−9^, Mann–Whitney *U* tests) per generation over diploids (Fig 2B). Interestingly, we observed a significant fitness difference between haploids of opposite mating types in both environments tested (Fig 2; rich medium *p* = 7.1 × 10^−10^, evolution conditions *p* = 0.013, Mann–Whitney *U* tests), suggesting a common underlying mechanism linked to mating type, rather than a specific mating type-by-environment interaction. Expression of the mating-type genes is costly [83], making components of this pathway common targets of adaptive loss-of-function mutations in haploids [7,67]. The observed fitness defect of *MAT***a** haploids in our experiments may reflect an expression burden imposed by the greater number of *MAT***a**-specific genes; a metabolic burden imposed by synthesizing the more complex, posttranslationally modified **a**-factor pheromone; or both. While previous large-scale studies in *S*. *cerevisiae*, *S*. *paradoxus*, and *S*. *eubayanus* have not reported general fitness differences between mating types of otherwise isogenic haploids [23,26], the subtle, but significant, differences we observed here may have been below previous limits of detection. Alternatively, the apparent defect of *MAT***a** cells may be specific to the 2 conditions we tested, so it remains to be determined whether our observations of mating-type fitness effects are completely generalizable in this strain background or more broadly. Irrespective of mating type, we find that haploids have a large and unexpected advantage over diploids under the ALE conditions.

### Haploid fitness advantage is primarily due to cell-type specification

**Fig 3:**
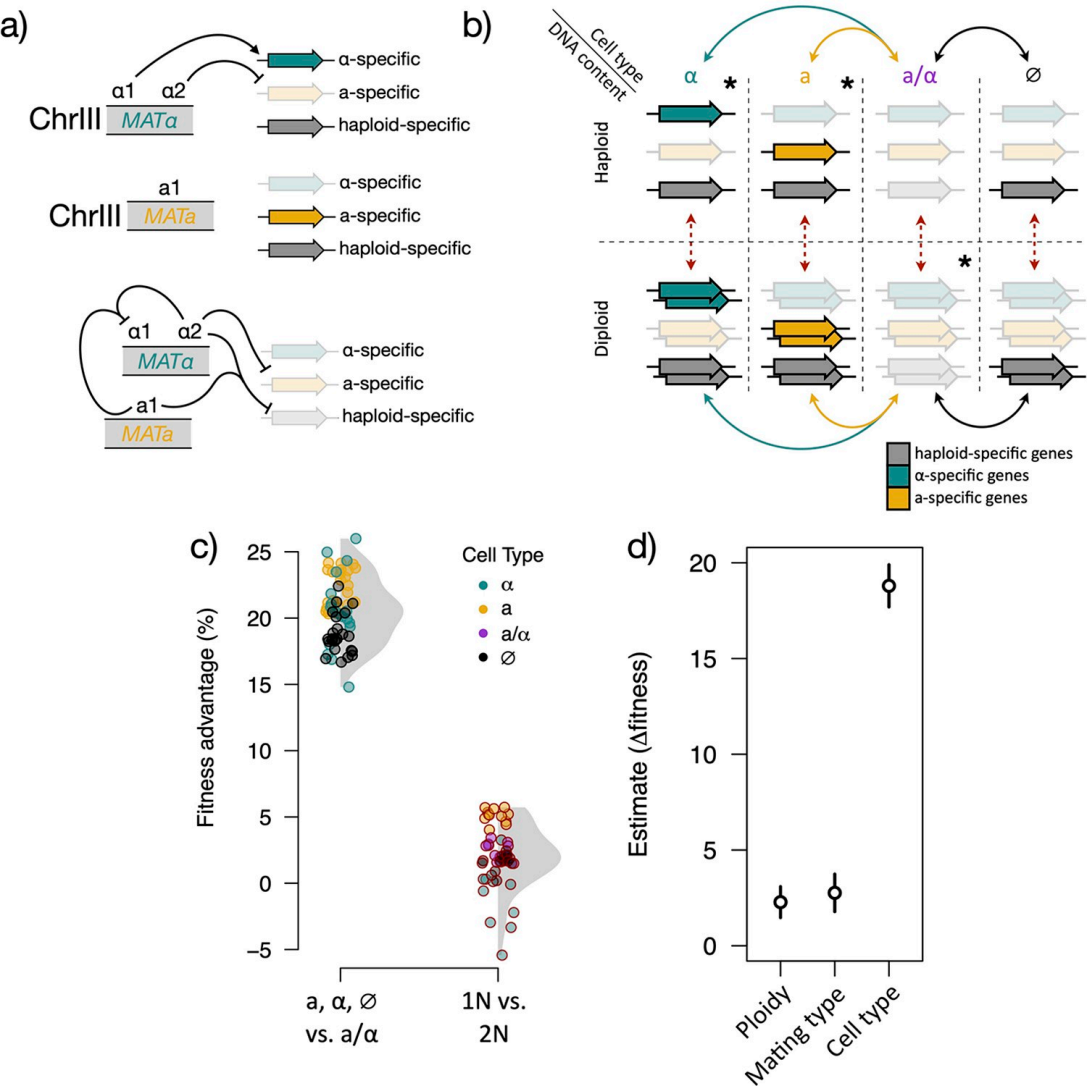
Cell-type specification is the primary contributor to adaptive fitness. (a) Simplified schematic of the cell-type specification circuit in *Saccharomyces* as determined by the *MAT* locus on ChrIII. Proteins encoded by each *MAT* idiomorph and their regulatory targets elsewhere in the genome are depicted. Haploids (top and middle) express mating type-specific genes and a common set of haploid-specific genes. Diploids (bottom) repress all 3 sets (transparent gene symbols). (b) Schematic of strains compared to determine ploidy and cell-type effects on fitness. The 3 classes of cell type-specific genes are depicted as colored bars, with opacity indicating expression in a given genotype. Asterisks in the upper right corner of fields indicate the 3 wild-type genotypes. The engineered genotypes (no asterisks) were created by deleting or adding complete *MAT* cassettes, with the exception of the “*MAT***-null**” strains (marked ∅), which express haploid-specific, but not mating type-specific, genes because they retain only *MATα2*. Dotted red lines are comparisons that show the effect of absolute DNA content, and solid lines are comparisons that show the effect of cell type, with colors corresponding to (c). (c) Points show differences in fitness in ALE conditions between genotypes that differ in only cell type (left cluster, e.g., **α** vs. **a**/**α**) or ploidy (right cluster). Gray shading shows the density distribution of each group. In each case, the wild-type state is taken as the baseline for comparison (diploid; **a**/**α** cell type). (d) Estimates and 95% confidence intervals for the effect of each variable on the difference in fitness. The data underlying this figure can be found in S1 Data. ALE, adaptive laboratory evolution.

In *Saccharomyces*, ploidy is intrinsically linked with cell- and mating-type specification, which are determined by the allelic composition of the *MAT* locus (Fig 3A) [28]. Some differences in cell physiology and gene expression patterns between ploidies are attributable solely to total cellular DNA content, while loss of heterozygosity at the *MAT* locus establishes one of 2 partially overlapping, cell type-specific gene expression programs [27,84,85]. The relationship between DNA content and cell-type specification can serve to confound inferences of the underlying basis of fitness differences between ploidies, although in limited cases, contributions of either absolute ploidy or *MAT* locus composition have been documented [7,23]. Here, we refer to “cell-type specification” as the distinction between genotypes with a full complement of cell-type master regulators at the expressed *MAT* locus (e.g., wild-type diploids containing *MATa1*, *MATα1*, *MATα2*) and those without. Cell types established by the absence of one or more cell-type regulators (e.g., wild-type haploids) effect the de-repression of a handful of genes, commonly referred to as “haploid-specific,” but whose expression is technically independent of ploidy and mating type.

To dissect the contributions of DNA content and cell type to organismal fitness in our system, we generated a panel of 8 otherwise isogenic genotypes with unique combinations of ploidy, mating type, and cell type-specific gene expression (Fig 3B). We measured the fitness of these strains in the ALE condition (Fig 3C) and estimated the separable effects of ploidy, mating-type specification, and cell type-specific gene expression patterns on fitness (Fig 3D). These 3 factors explained the majority of the variance in measured fitness across genotypes (multiple R^2^ = 0.96, df = 86, *p* < 2.2 × 10^−16^), with each having a significant effect (*p* ≤ 2.56 × 10^−7^). Remarkably, cell-type specification had an impact on organismal fitness that was almost an order of magnitude greater than either ploidy or mating type (Fig 3D, fitness advantage estimate 18.8%, 95% CI: 17.7, 19.9), and explained far more of the variance (proportion sum squares: cell type, 0.93; ploidy, 0.016; mating type, 0.014). Absolute ploidy nonetheless impacted fitness across cell types, with haploids experiencing a 2.3% advantage relative to diploids in the ALE condition (Fig 3D, 95% CI: 1.5, 3.1). Paradoxically, expression of mating type-specific genes in these experiments appeared to modestly increase fitness between haploid-like cell types in the ALE condition (Fig 3D), in contrast to the documented cost of their expression in other conditions [83]. While one possible interpretation is that both sets of mating type-specific genes confer bona fide fitness advantages to cells growing in maltose medium, an alternative explanation for this apparent discrepancy is that haploid-like, *MAT***-null** cells experience modest fitness defects as a result of their aberrant and artificial cell type. As such, our analyses may slightly underestimate the fitness benefit attributable to haploid-like cell type in the ALE condition. We conclude that the cell type specified by the *MAT* locus, rather than absolute ploidy per se, has the largest effect on fitness in the ALE condition.

### Dynamics of other ploidy variants in adapting populations

We next investigated the evolutionary dynamics and adaptive benefit of the other shared ploidy variant in the evolved clones: aneuploidy of ChrXV (Fig 1B). We performed bulk whole-genome sequencing on the cryopreserved replicate ALE populations from the same time points at which we assayed ploidy states by flow cytometry (S2B Fig) and quantified the apparent frequency of each chromosome in both populations as estimated by relative coverage (S4 Fig). The only aneuploidies that rose to an appreciable frequency were those sampled in our clonal isolates: ChrXV (both populations) and ChrI (1 population). The dynamics of ploidy variants—including both aneuploidy and haploidy—and the relative timing of their emergence differed between populations. In the first population, the ChrXV aneuploidy rose to near fixation prior to the apparent emergence of haploids, while the rise in frequency of the ChrI aneuploidy was approximately coincident with that of haploids (S4B Fig). In the second population, the ChrXV aneuploidy and the haploid state had remarkably similar trajectories, with ChrXV aneuploidy appearing to precede haploidy slightly: both rose precipitously in frequency after approximately 50 generations, declined dramatically, and subsequently rebounded by the terminal time point (S4B Fig). These results suggest that a haploid lineage with ChrXV aneuploidy was subject to clonal interference from 1 or more highly fit genotypes in this replicate.

The change in frequency of the ChrXV aneuploidy over time in both populations suggested a strong fitness benefit, which we reasoned was likely attributable to the presence of *AGT1* on this chromosome (Fig 1B). *S*. *eubayanus* Agt1p is a homolog of the well-characterized *S*. *cerevisiae* α-glucoside transporter, but in contrast to canonical *MAL* gene clusters that contain structural and regulatory maltose metabolism genes, *S*. *eubayanus AGT1* is isolated in the subtelomeric region of ChrXV. In our genome assembly, no predicted genes intersperse the *AGT1* start codon and the beginning of telomeric repeats some 6,770 bp upstream. We did not identify any homologs of genes encoding *MAL* regulators, transporters, α-glucosidases, or isomaltases on ChrXV, nor other strong candidates to explain the adaptive potential of the aneuploidy. We thus tested whether copy number variation at *AGT1* alone provided an adaptive benefit in the ALE environment to explain the sweep of ChrXV aneuploidy in both populations. We inserted an additional copy of *AGT1* under its native promoter and terminator into the genomes of diploids and haploids at a separate site, and we measured the fitness of the resulting strains in the ALE conditions. As predicted, increased *AGT1* copy number conferred a substantial and significant fitness benefit in both diploids and haploids (S5 Fig). Haploids received a more modest increase in fitness than diploids upon the addition of *AGT1* (S5A Fig), which we attribute to the effects of diminishing returns epistasis; nonetheless, they were significantly more fit overall (S5B Fig). There was no interaction between the haploid mating type and the fitness effect of increased *AGT1* copy number. Thus, the ChrXV aneuploidy we observed in both clonal isolates and the ALE populations likely contributed to adaptation by increasing copy number of *AGT1*, and its emergence may have preceded that of haploids.

### *AGT1* expression is elevated in aneuploid haploids

**Fig 4:**
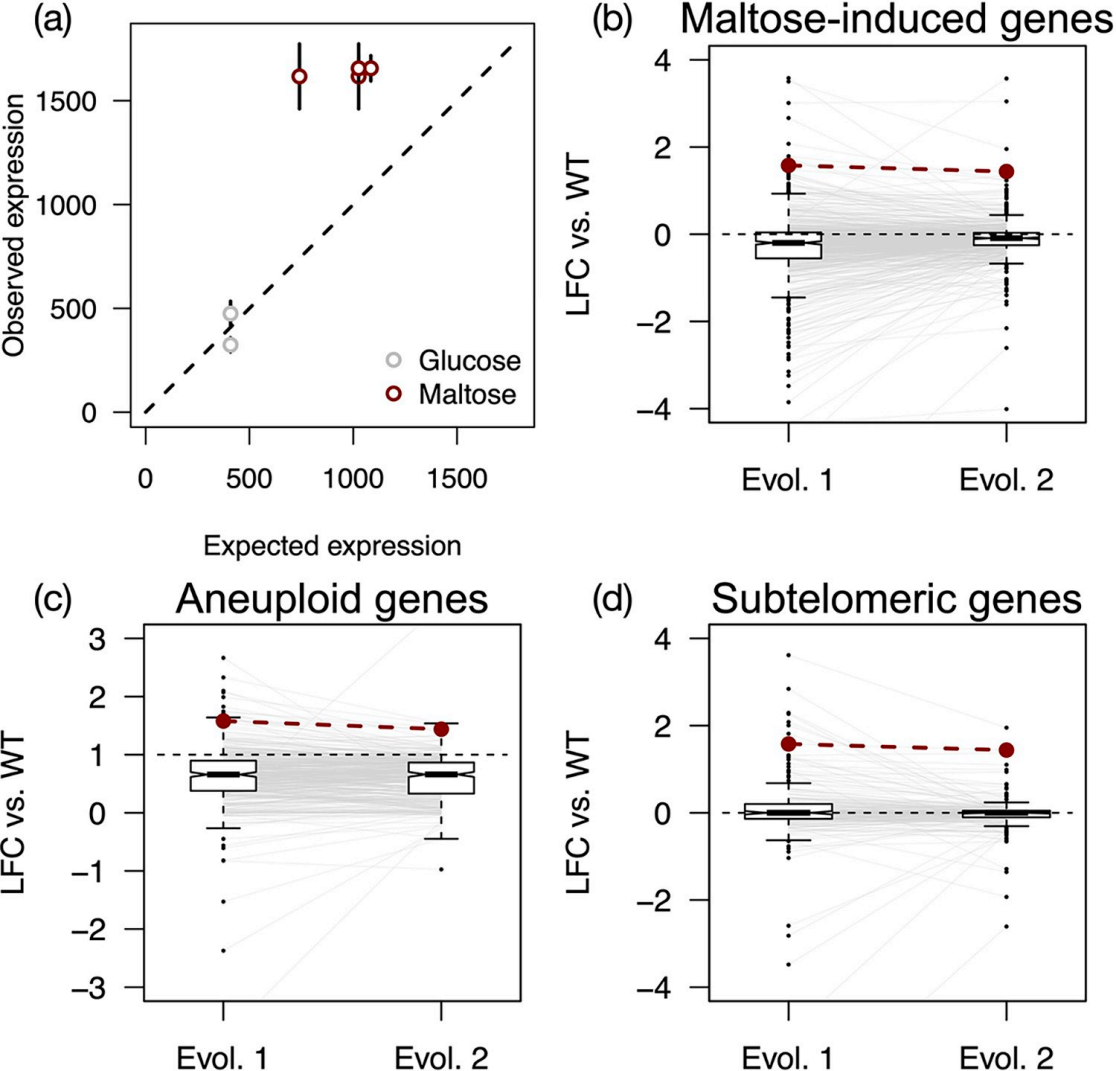
Increased expression of an α-glucoside transporter gene in haploids. (a) Points and bars show mean and standard error of *AGT1* expression in evolved haploids, plotted against the null expectation of expression based on copy number variation and induction in the WT strain. Expression in maltose is greater than the null expectations in the evolved haploids (*p* = 0.0005, one-sided Mann–Whitney *U* test). (b–d) Boxplots show LFC of gene expression in maltose in evolved haploids compared to the WT strain. Whiskers extend to 1.5× the interquartile range. Lines connect the y-axis coordinates of the same gene in each evolved isolate; axes are scaled such that an occasional outlier is truncated from the plot space for a single strain. *AGT1* expression is plotted as red dots and lines, and black dashed lines indicate the null expectation for expression values. (b) Genes induced in maltose in the WT strain (*n* = 544). (c) Genes on aneuploid ChrXV (*n* = 370). (d) Subtelomeric genes (*n* = 200). For all classes, expression in either evolved haploid is not significantly greater than the null expectation (one-sided *t* tests, min. *p* = 0.42). The data underlying this figure can be found in S1 Data. LFC, log2-transformed fold change; WT, wild-type.

The conditional fitness advantage of haploids and increased fitness of haploid-like cell types suggested an unexpected regulatory link between maltose metabolism and haploid-specific genes (i.e., those genes de-repressed in the absence of a heterozygous *MAT* locus). To identify potential targets of this interaction, we analyzed mRNA-seq data collected from the wild-type diploid and evolved haploids grown in conditions mimicking the evolution experiment (SC-maltose), as well as a baseline for comparisons (SC-glucose). Although the haploid strains had discrete polymorphisms, they shared a common cell type and aneuploidy of chromosome XV (Fig 1B); thus, we reasoned that common differences in expression between these isolates relative to the wild-type strain should be attributable to one (or both) of these shared genotypes. Transcriptomes of the evolved haploids were highly similar, as expected (S6 Fig). Differentially expressed genes (DEGs) between the wild-type strain and evolved haploids were enriched for cell and mating type-specific transcripts and genes on aneuploid chromosomes; however, there was no clear functional enrichment among DEGs to explain the maltose-specific haploid fitness advantage. The *AGT1* transporter on ChrXV was the single maltose metabolism gene up-regulated in maltose in both evolved haploids when compared to the wild-type strain, which was expected given its 2-fold relative copy number in these isolates (Fig 1B). Upon closer examination, however, *AGT1* expression was higher than the 2-fold increase expected commensurate with its relative copy number [86,87]. Indeed, *AGT1* expression in haploids exceeded null expectations based on 2 distinct models (Fig 4A): (1) we calculated the fold change for *AGT1* in the ancestral strain in maltose compared to glucose and applied this multiplier to the glucose expression level in the evolved haploids; and (2) we applied a 2-fold multiplier to the gene expression levels in the wild-type strain in both glucose and maltose, which accounted for copy number variation in the evolved haploids. While *AGT1* expression in glucose in the evolved haploids was in line with the naïve aneuploid expectation (*p* = 0.81, one-sided Mann–Whitney *U* test), its expression in maltose in the evolved haploids was an average of 69% higher than could be modeled by accounting for copy number and native regulation (Fig 4A, *p* = 0.0005, one-sided Mann–Whitney *U* test).

We next asked whether increased *AGT1* expression could be explained by subtle changes in global gene expression levels between the wild-type diploid and evolved haploids. We compared expression levels of 2 relevant classes of genes under which *AGT1* falls and which we reasoned might be subject to modest differential expression: maltose-induced genes and subtelomeric genes (Fig 4B and 4D). We also examined expression of genes on the aneuploid ChrXV to test whether these broadly exceeded the expectation of a 2-fold expression increase commensurate with copy number (Fig 4C). In each case, expression in the evolved haploids was not significantly greater than the null expectation (one-sided *t* tests, *p* > 0.4), and *AGT1* expression in maltose was in the upper tail of gene expression values for each class. Most notably, *AGT1* expression in maltose relative to the euploid diploid ranked higher than 95.9% and 98.6% of other ChrXV genes in each evolved haploid, respectively.

Consistent with numerous studies in yeasts, we observed average expression from the aneuploid ChrXV to be elevated, if not exactly 2-fold higher than in the euploid diploid [86,88–93]. Importantly, we observed this expression attenuation across conditions, meaning that the elevated expression observed at *AGT1* is not likely to be an artifact of condition-specific aneuploid gene expression differences. Compared to the wild-type strain, we observed median fold changes for ChrXV genes of 1.58 in maltose for both evolved haploids (Fig 4C) and 1.72 and 1.70 in glucose, respectively (S7A Fig). Indeed, the potential effect of cell type on *AGT1* expression becomes even more evident in light of the median expression levels of aneuploid genes in haploids: *AGT1* is up-regulated an average of 4-fold in maltose across the haploid strains (S4 Table and S7B Fig), while median fold changes for all ChrXV genes between maltose and glucose are 0.969 and 0.970 for each haploid, respectively (S7B Fig). Compared to approximately 2.3-fold induction of *AGT1* in maltose in the euploid diploid (S4 Table), this increased induction in the evolved isolates may reflect the combinatorial effects of cell type and sugar response. As increased *AGT1* copy number (which should result in a concomitant increase in expression) significantly increases fitness in maltose (S5 Fig), the increased expression observed in haploids is also likely to contribute to adaptation—and could explain the condition-specific fitness advantage of isogenic haploids.

Naturally, it remains a possibility that the elevated expression of *AGT1* that we observed in aneuploid haploids is the result of an interaction (whether direct or indirect) between this locus and one or more genes elsewhere on the chromosome. In addition, aneuploidy itself can trigger a transcriptional response [89,92,94], although to our knowledge, this response does not extend to maltose metabolism genes. As a complete dissection of aneuploidy response and its targets in *S*. *eubayanus*—as well as how this response may differ between ploidies and cell types—is beyond the scope of the current work, we instead investigated potential regulators of *AGT1* that could explain its cell type-linked increase in expression (detailed below). Thus, our data cannot unequivocally reject the hypothesis that whole-chromosome duplication itself may affect *AGT1* expression in addition to the role we have established for gene copy number and cell type.

### The *AGT1* promoter integrates cell-type and sugar-responsive regulatory networks

**Fig 5:**
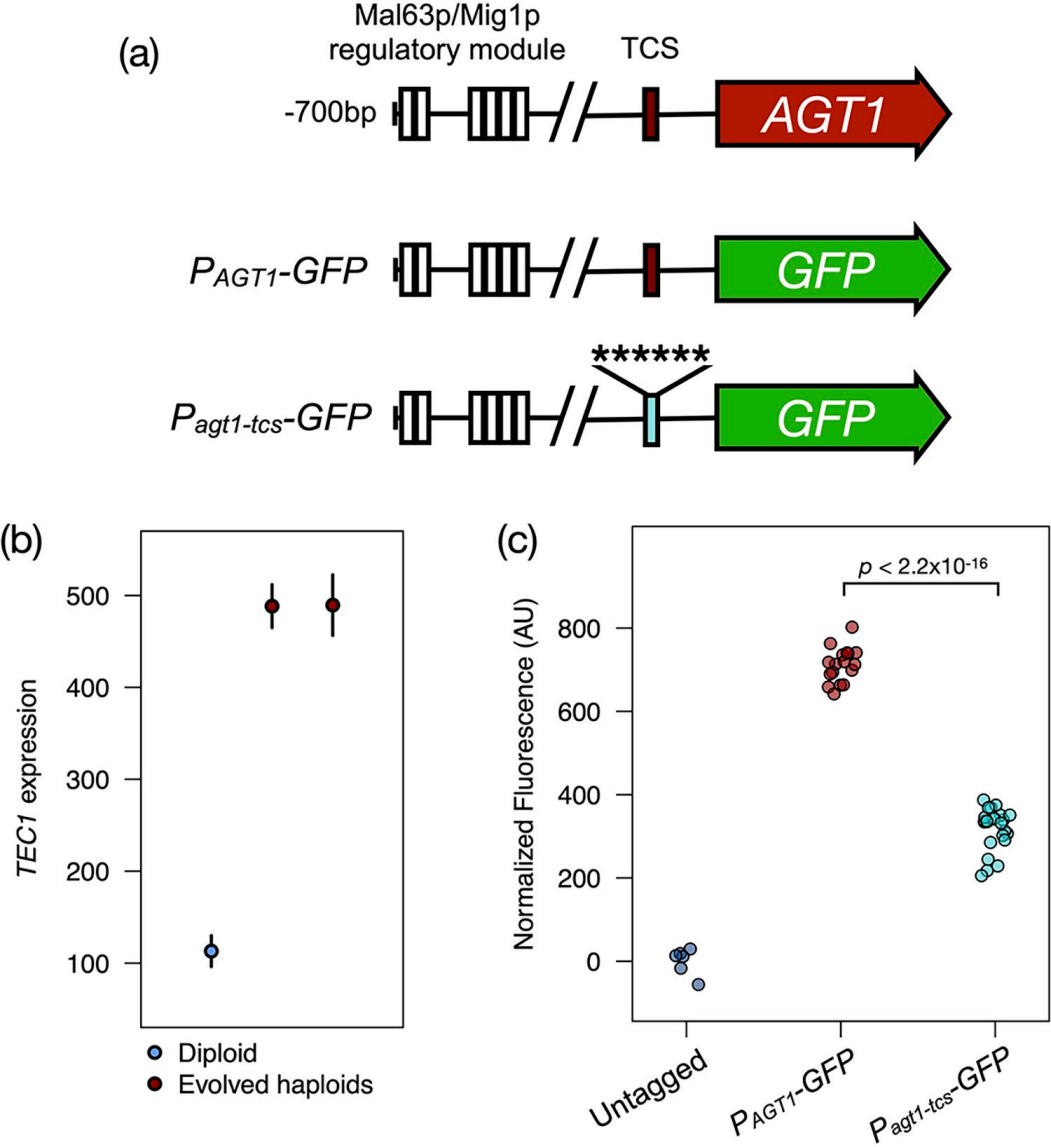
*AGT1* is regulated by cell type. (a) Schematic of the *AGT1* promoter and reporter constructs. A clustered regulatory module containing Mal63p and Mig1p motifs (white boxes) lies upstream of *AGT1* in its native context (top), which is reminiscent of other maltose metabolism genes in *Saccharomyces*. One further predicted binding site for each regulator that lies closer to the coding sequence is omitted for space. The promoter contains a motif for Tec1p (TCS, red box). We generated reporter constructs expressing *GFP* from the wild-type promoter (middle, *P*_*AGT1*_) and a version with point mutations to the Tec1p motif (bottom, *P*_*AGT1-tcs*_). (b) *TEC1* expression is cell type dependent in *S*. *eubayanus*. Points and bars show mean and standard error of *TEC1* expression (normalized counts) in the wild-type diploid and evolved haploids, averaged across conditions. (c) Point mutations to the predicted Tec1p-binding site in the *AGT1* promoter reduce reporter expression. Each point shows the mean population fluorescence for a replicate experiment with a control untagged strain (gray), as well as strains expressing GFP from the wild-type *AGT1* promoter (red) or a promoter with a mutated Tec1p motif (teal). All engineered strains are significantly different from the untagged control (*p* ≤ 4.3 × 10^−6^, two-sided *t* tests), and groups of promoter genotypes differ significantly (two-sided *t* test). The data underlying this figure can be found in S1 Data. TCS, TEA/ATTS consensus sequence.

We first investigated potential regulators of *AGT1* by scanning its promoter for putative transcription factor-binding sites using high-confidence *S*. *cerevisiae* motifs (S5 Table). This analysis identified clustered binding motifs for the canonical positive and negative regulators of maltose metabolism genes, Mal63p and Mig1p (Fig 5A), in an organization consistent with the characterized regulatory module that controls the expression of maltose metabolism genes in *S*. *cerevisiae* [95–97]. Although a causal relationship has not been directly established, the presence of Mal63p consensus sequences upstream of maltose metabolism genes is well correlated with their induction by maltose in the type strain of *S*. *eubayanus* [58]. In addition to these expected regulators, we identified putative binding sites for several transcription factors that are involved in regulating filamentous growth (e.g., those encoded by *ASH1*, *SIP4*, *STE12*, *FKH1*, *MIG1/MIG2*, and *NRG1*). This category was particularly noteworthy because filamentous growth can be induced in response to glucose depletion as a starvation response, and it requires a haploid-specific gene, *TEC1* [27,98–103]. In addition to dimerizing with Ste12p, Tec1p can activate target genes as a monomer in a dosage-dependent fashion [104–106], and it has been experimentally mapped to its consensus motif (TEA/ATTS consensus sequence or TCS) in vivo across the genus *Saccharomyces* [107]. We identified a TCS in the promoter of *AGT1* and hypothesized that Tec1p could mediate the cell type-specific increase in *AGT1* expression we observed in haploids. Supporting this notion, and consistent with its characterization as a haploid-specific gene in *S*. *cerevisiae* [27], *TEC1* was significantly up-regulated in both evolved isolates in our dataset (Fig 5B and S4 Table).

To test this hypothesis, we cloned *yEGFP* under the control of the wild-type *AGT1* promoter (*P*_*AGT1*_), as well as a promoter variant with point mutations in the predicted Tec1p-binding site (*P*_*agt1-tcs*_), and introduced a single copy of these reporters to the genome of euploid *MAT***a** haploids. We then measured single-cell fluorescence of the resulting strains grown in maltose by flow cytometry. Mutation of the Tec1p-binding site significantly decreased fluorescence from the reporter construct compared to the wild-type promoter (*p* < 2.2 × 10^−16^, two-sided *t* test), but it did not abolish expression completely (Fig 5C). These results are consistent with the expression data and collectively suggest that *AGT1* receives regulatory input from both cell-type and sugar-responsive networks, with separable activation by Tec1p and induction in the presence of maltose. We also measured expression of the *P*_*AGT1*_*-GFP* reporter in several media conditions using a less sensitive plate-based assay (S8 Fig). During growth on glucose and galactose—both expected to be non-inducing—no fluorescence above baseline was detected. Growth on maltose induced expression significantly, as did growth on methyl-*α*-glucoside, another substrate transported by *AGT1* in *S*. *cerevisiae* [108,109]. Interestingly, we also observed modest reporter expression when cells were pre-grown in glucose and switched to medium containing no sugar (S8 Fig), further supporting the notion that *AGT1* may be expressed in response to suboptimal carbon conditions in this background.

In synthesis, the evidence for a direct fitness advantage by haploid-like cell types (Figs 2 and 3), increased expression of fitness-modifying *AGT1* in haploids (Fig 4), and the partial dependence of *AGT1* expression on the motif for a haploid-specific transcription factor (Fig 5) suggests a relationship between ploidy evolution and adaptation in our system. Future experimentation could more clearly define the role of *AGT1* and its regulation in definitively driving the ploidy evolution we observed, such as by replaying the experimental evolution using genotypes lacking *TEC1* or with different promoters driving *AGT1*. Although absolute ploidy does seem to impart a small fitness difference in our experiments, the full impact on fitness requires cell type (Fig 3C and 3D).

## Conclusions

Resolving the genotype-to-phenotype map remains a central goal in genetics and evolutionary biology, but it has frequently proven challenging, even in microbes. While gene content is generally correlated with metabolic traits across budding yeasts [25], regulatory nuances in organisms that are not traditional models can confound inferences of phenotypes from genome sequences [63,110,111]. In the taxonomic type strain of *S*. *eubayanus*, structural maltose metabolism genes in canonical *MAL* clusters are exquisitely repressed or induced hundreds-fold in response to carbon source [58], which is similar to their *S*. *cerevisiae* homologs [112]. By contrast, in the strain from the Holarctic subpopulation studied here, what appears to be the focal maltose transporter is partially decoupled from such stringent catabolite regulation: *AGT1* is only induced approximately 2.3-fold in the wild-type strain in maltose (S4 Table). We can envision 2 potential explanations for the apparently unusual regulation of this gene.

First, *AGT1* is likely to encode a transporter with broad substrate affinity like its *S*. *cerevisiae* homolog [64,113–116], whereas other phylogenetically distinct maltose transporters tend to have higher specificity [108,117]. It is possible that selection favored placing control of this generalist transporter under a broader transcriptional response to starvation or glucose depletion as part of a scavenging strategy, which the transition to filamentous growth is thought to represent [102]. Indeed, recent work has suggested that maltose may be an unexpected inducer of filamentous growth in *S*. *cerevisiae* [118]. Decoupling alternative carbon metabolism genes from their stringent canonical regulation has also been shown to be adaptive among isolates of *S*. *cerevisiae* subject to specific ecologies [119] and ALE in fluctuating environments [120].

Second, the organization—and potentially regulation—of *AGT1* in *S*. *eubayanus* may be reflective of the ancestral state for *Saccharomyces*. In strains of *Saccharomyces paradoxus*, *Saccharomyces mikatae*, and *S*. *eubayanus*, *AGT1* homologs are scattered in subtelomeric regions and not in canonical *MAL* loci, while homologs encoding high-affinity maltose transporters tend to occur in gene clusters with the typical organization [63,96]. Thus, the specific organization of *AGT1* in the *MAL1* locus of model *S*. *cerevisiae* strains—and its resulting exquisite regulation by glucose and maltose—could itself represent a derived state that is not reflective of wild yeasts. Indeed, there is clade-specific variation within *S*. *cerevisiae* as to whether the *MAL1* locus is occupied by the generalist *AGT1* or a gene encoding a high-specificity maltose transporter [121], suggesting that domestication may have shaped the genetic architecture of α-glucoside metabolism in this model eukaryote [122]. Supporting this notion, *AGT1* homologs can be readily detected in publicly available *Saccharomyces* genomes, while growth on maltotriose—a sugar transported by *AGT1* but not most other maltose transporters—is extremely rare [62]. A notable exception is *Saccharomyces jurei*, the first wild *Saccharomyces* reported to grow on maltotriose, which contains a clear homolog of *AGT1* that requires extensive starvation or depletion of fermentable carbon sources for its induction [123]. Whether the organization and regulation of *AGT1* in Holarctic *S*. *eubayanus* represent a derived or ancestral state, it created a paradigm wherein a transition between ploidy states—and thereby cell types—was the adaptive step conferring the greatest increase in fitness among evolved genotypes we tested. The precise mutational event or events underlying this adaptive step remain unclear; however, with estimates of the rate of single-chromosome loss in *S*. *cerevisiae* ranging from roughly 10^−4^ to 10^−8^ per generation [8,124–127], sequential loss of 14 to 15 chromosomes is highly improbable in our system. A programmed transition from diploid to haploid during meiosis is an integral part of the budding yeast life cycle; thus, we suspect that rare sporulation events—perhaps triggered by the cycling nutrient availability in our batch-style ALE—enabled haploids to arise.

Remarkably, there is a strong parallel to the rewiring of carbon metabolism to cell type control in certain domesticated strains of *Saccharomyces cerevisiae*. Diastatic strains (sometimes called *Saccharomyces cerevisiae* var. *diastaticus*) are characterized by their hyperattenuation, which is attributable to the presence of a novel extracellular glucoamylase, encoded by *STA1* [128]. *STA1* is a chimeric gene, created by the fusion of the sporulation-specific intracellular glucoamylase gene *SGA1* with the promoter and portions of the coding sequence of *FLO11*, which encodes a flocculin involved in filamentous growth that is subject to cell type-specific regulation [129–134]. Due to this gene fusion, *STA1* is expressed in a cell type-specific manner, and its regulation integrates catabolite repression by glucose and direct activation by Tec1p [135–137]. The cell type dependence mediated by Tec1p in this case may have caused selection for haploidy among diastatic strains of the Beer 2 clade of *S*. *cerevisiae* [15,138], which lack the clade-specific *AGT1* allele at the *MAL1* locus [121] and therefore must hydrolyze higher-order maltodextrins extracellularly.

*STA1* in diastatic brewing strains, *AGT1* in our ALE strains, and genes related to pathogenesis across fungi have undoubtedly experienced intense bouts of selection, and it seems that ploidy and cell type changes may be a common means of adapting, at least in microbial eukaryotes that have this flexibility. Here, we have shown that striking, rapid, and unusual ploidy evolution in a wild yeast is associated with the integration of regulatory inputs from metabolism and cell-type networks at the *AGT1* promoter. Our results thus provide compelling insight into the basis of a ploidy fitness effect in fungi.

How generalizable might these principles be? Given the evolutionary lability of ploidy, its link to cell type, and evidence for interactions between cell type and conditionally adaptive traits in other fungal systems, we propose that environment- and genotype-specific regulatory nuances might play a broad role in shaping both the extant diversity of fungal ploidy states and the conflicting, and often cryptic, ploidy and cell-type evolution seen in systems experiencing intense selection. This view argues that interactions between cell types, ploidy states, and conditionally adaptive traits may be common during fungal evolution and may influence fungal life cycles more than is currently appreciated.

## Materials and methods

### Strains, plasmids, and cultivation conditions

Strains, oligonucleotides, and plasmids used in this work are listed in S1 and S2 Tables. Yeast strains were propagated on rich YPD medium (10 g/L yeast extract, 20 g/L peptone, 20 g/L glucose, with 18 g/L agar added for plates), Synthetic Complete medium with maltose or glucose (5 g/L ammonium sulfate, 1.7 g/L Yeast Nitrogen base, 2 g/L drop out mix, 20 g/L maltose or glucose, pH = 5.8, with 18 g/L agar added for plates), or Minimal Medium (5 g/L ammonium sulfate, 1.7 g/L Yeast Nitrogen base, 10 g/L maltose or glucose, pH = 5.8) at room temperature. Yeast strains and ALE populations were stored in 15% glycerol at −80° for long-term storage. For supplementation with drugs, 1 g/L glutamic acid was substituted for ammonium sulfate in SC media. G418, Hygromycin B, and Nourseothricin (CloNAT) were added to media at final concentrations of 400 mg/L, 300 mg/L, and 50 mg/L, respectively. Transformation of *S*. *eubayanus* was performed via a modified PEG-LiAc method [139] as previously described [64]. Repair templates for homologous recombination were generated by PCR using Phusion polymerase (NEB) and purified genomic DNA as template or Taq polymerase (NEB) and purified plasmid as template per the manufacturer’s instructions, followed by purification with QiaQuick or MinElute spin columns (Qiagen). For CRISPR-mediated transformations, pXIPHOS vectors [111] expressing Cas9 and a target-specific sgRNA were co-transformed into strains with double-stranded repair templates. Multi-fragment repair templates were assembled by overlap extension PCR with Phusion polymerase or co-transformed as multiple linear fragments with 80 bp overlapping homology for in vivo recombination. Following transformation, yeast cells were plated to YPD for recovery and replica-plated to medium containing the appropriate antibiotic for selection after 24 to 36 h. Gene deletions and knock-ins were verified by colony PCR and Sanger sequencing.

Plasmids were propagated in *E*. *cloni* 10G cells (Lucigen) and purified using the ZR miniprep kit (Zymo Research). sgRNAs for CRISPR/Cas9-mediated engineering were designed using CRISpy-pop [140], obtained as single-stranded 60-mers from Integrated DNA Technologies, inserted into *Not*I-digested pXIPHOS vectors using HiFi assembly (NEB), and verified by Sanger sequencing.

### Growth assays

Strains were streaked to single colonies on solid YPD agar, and individual colonies were inoculated to 250 μL YPD in flat-bottom 96-well plates for preculturing in a randomized layout. Precultures were incubated for 3 days at room temperature, serially diluted in Minimal Medium, and inoculated to Minimal Medium containing maltose or glucose at a final dilution factor of 10^−4^. Plates were incubated on a SPECTROstar Omega plate reader (BMG Labtech) equipped with a microplate stacker, and OD_600_ was measured every hour. Raw plate reader data were processed using GCAT [141] and further analyzed in R v4.0.4 (https://www.R-project.org) [142].

### *MAT* locus genotyping

We used a multiplex colony PCR with Taq polymerase (NEB) and oligos oHJC120, oHJC121, and oHJC122 to genotype the *MAT* locus of strains following tetrad dissection, mating type engineering, and for estimating the frequency of haploids in ALE populations after plating. The multiplex reaction gives rise to *MAT***a**- and *MAT***α**-specific amplicons of differing size, which were resolved on 2% agarose gels. All reaction conditions were per the manufacturer’s instructions and were carried out alongside controls (diploid *MAT***a**/*MAT***α**; haploid *MAT***a**; haploid *MAT***α**; no input DNA). We discarded any experiment where the controls did not produce the expected amplicons (or lack thereof). To estimate the frequency of haploids in populations, we screened a total of 55 to 56 single colonies across 4 independent platings of each population. We note that this approach cannot formally distinguish between cells of different ploidies with rare aberrant *MAT* locus composition (e.g., diploid *MAT***a**/*MAT***a** will generate the same amplicon pattern as haploid *MAT***a**; loss of *MAT* locus heterozygosity in diploid *S*. *cerevisiae* has been estimated to occur at a rate of 2 × 10^−5^ per cell per generation [20]). In addition, this *S*. *eubayanus* background is homothallic, meaning that any diploid colony recovered following plating might represent a haploid cell in the experimental population maintained in liquid medium. The rate of mating type switching and clone-mate selfing on solid medium is likely orders of magnitude higher than loss of *MAT* locus heterozygosity [14,143]; thus, our PCR-based estimates of haploid frequency may be conservative.

### Mating type testing

In addition to molecular validation of engineered strains, we tested the expressed mating type of strains with altered *MAT* locus composition using microbiological assays. To assess *MAT***α** expression, a saturated liquid culture of *S*. *cerevisiae bar1-Δ* was diluted 100-fold and spread-plated to YPD, and 10 μL of overnight query strain culture was spotted on top. For *MAT***a** expression validation, saturated cultures of query strains were diluted 100-fold and spread-plated to YPD, and a disc of sterile filter paper saturated with 10 μL of 200 μm **α**-factor (Zymo Research) was gently embedded in the center. Every experiment included wild-type controls of known mating type (diploid *MAT***a**/*MAT***α**; haploid *MAT***a**; haploid *MAT***α**), and in each case, growth inhibition by **α**-factor or of *S*. *cerevisiae bar1-Δ* was scored relative to controls and compared to the parental strain, where applicable.

### DNA sequencing

To obtain high molecular weight genomic DNA from wild-type strain yHRVM108, 2 single colonies were each inoculated in 90 mL YPD and grown to mid-log phase (OD_600_ = 0.5), harvested by centrifugation, washed with water, and resuspended in 5 mL DTT buffer (1 M sorbitol, 25 mM EDTA, 50 mM DTT). Cells were DTT-treated for 15 min at 30° with gentle agitation, pelleted, washed with 1 M sorbitol, and resuspended in 1 mL 1 M sorbitol with 0.2 mg 100T Zymolyase. Cells were spheroplasted for 30 min at 30° with gentle agitation, then pelleted. The pellet was gently resuspended in 450 μL EB (Qiagen) without pipetting and treated with 50 μL RNAse A (10 μg/mL) for 2 h at 37°, and 55 μL 10% SDS was added, and the mixture was incubated for a further hour at 37° with gentle agitation to lyse spheroplasts. DNA was extracted by the phenol/chloroform method and precipitated by addition of 1 mL 100% ethanol and overnight incubation at −80°. Precipitated DNA was pelleted, washed twice with 70% ethanol, dried briefly, and gently resuspended in 100 μL TE buffer at room temperature without pipetting for 2 h. DNA was quantified using the Qubit dsDNA BR kit (Thermo Fisher Scientific), and purity was assessed by Nanodrop (Thermo Fisher Scientific).

DNA concentration was adjusted to 50 ng/μL, and 7.5 μg genomic DNA was subjected to SPRI size selection with Agencort AMPure XP beads in custom buffer following the recommended protocol from Oxford Nanopore Technologies; 1 μg size-selected DNA was prepared for sequencing using the SQK-LSK109 ligation kit (Oxford Nanopore Technologies), and approximately 40 fmol library was loaded on a single FLO-FLG001 flowcell. Basecalling was performed with Guppy v3.2.1. ONT sequencing yielded 885.1 Mb of base-called reads passing quality filtering, for approximately 74-fold genomic coverage.

We prepared genomic DNA for Illumina sequencing from the wild-type strain and evolved isolates as described previously [25]. Strains were streaked to single colonies, and colonies were inoculated to 3 mL YPD medium and grown to saturation before collection for DNA extraction. Purified DNA was quantified by Qubit dsDNA BR assay (Thermo Fisher Scientific), and purity and quality were assessed by Nanodrop (Thermo Fisher Scientific) and agarose gel electrophoresis. Library preparation and Illumina sequencing of the wild-type strain and clonal evolved isolates were performed by the DOE Joint Genome Institute. Paired-end libraries were sequenced on a NovaSeq S4 with 150 bp reads, yielding an average of 8.47 million reads per sample. For the wild-type strain yHRVM108, we also integrated publicly available reads (SRA: SRX1317977) from a previous study [49].

To track the frequency of aneuploidies in the ALE populations, entire populations cryopreserved at −80°C in 15% glycerol were gently thawed, and 20 μL was inoculated directly to 2 mL SC-2% Maltose and grown to saturation. DNA was extracted as described above, and libraries were prepared using the NEBNext Ultra II FS kit (New England Biolabs) per the manufacturer’s instructions. Libraries were sequenced on an Illumina NovaSeq at the University of Wisconsin-Madison Biotechnology Center with paired-end 150 bp reads, yielding an average of 2.23 million reads per sample. All raw reads were processed using Trimmomatic v0.3 [144] to remove adapter sequences and low-quality bases.

### Genome assembly, annotation, and analysis

Canu v1.9 [145] was used to generate a genome assembly with Nanopore sequencing reads from the wild-type strain, which was subsequently polished with Illumina reads using 3 rounds of Pilon v1.23 [146]. The genome assembly was annotated using the Yeast Genome Annotation Pipeline [147]. We mapped each predicted gene to its *S*. *cerevisiae* homolog using BLASTp v2.9 [148]. QUAST v5.0.2 [149] and BUSCO v3.1.0 [150] were used to assess genome completeness, and chromosomes in the assembly were assigned numbers corresponding to the *S*. *eubayanus* type strain reference genome [58,151] using MUMmer v3.2.3 [152] and BLASTn v2.9. BWA v0.7.12 [153] and samtools v1.9 [154] were used to map short reads from all sequenced strains and population samples to the assembly, and BEDtools v2.27 [155] was used to call sequencing depth. Coverage across the genome of each strain or population was analyzed in R and assessed by manually inspecting coverage plots of each chromosome. Final genome-wide Illumina-sequencing depths for each strain were 200.7-fold (wild-type), 106.1-fold (evolved clone 1), and 84.2-fold (evolved clone 2); sequencing depths for population samples ranged from 14.3- to 109.3-fold (median: 34.3). We used FreeBayes v1.3.1 [156] to call variants in each strain, requiring a minimum coverage depth of 10 to report a position, and manually inspected putative variants in IGV [157]. To annotate predicted transcription factor binding sites in the promoter of *AGT1*, we used the 700 bp upstream of the start codon as a query for YEASTRACT+ [158].

### RNA extraction

Strains were streaked to singles on solid YPD agar, colonies were precultured to saturation in synthetic complete medium with 2% glucose or maltose as the sole carbon source, and precultures were back-diluted into the same medium at a low initial OD_600_ before being harvested in early log phase, a growth regimen designed to fully alleviate catabolite repression of alternative carbon metabolism genes. Cells were harvested by centrifugation at 4°C after the addition of 0.1 volumes of 5% acid phenol/95% ethanol, and pellets were flash-frozen. RNA was extracted using the hot acid phenol/ethanol precipitation method, with the addition of glass beads during vortexing to aid lysis efficiency. Samples were treated with RQ1 DNAse (Promega) followed by a final purification by RNeasy column (Qiagen). RNA yield and quality were assessed by Qubit BR RNA assay (Thermo Fisher Scientific), agarose gel electrophoresis, Qubit RNA IQ assay (Thermo Fisher Scientific), and Nanodrop.

### RNA sequencing and analysis

mRNA enrichment, library preparation, and Illumina sequencing were performed by the DOE Joint Genome Institute for biological triplicate samples for each strain and condition. Paired-end libraries were sequenced on a NovaSeq S4 with 150 bp reads, yielding an average of 23.23 million reads per sample. Raw mRNA-seq reads were processed with BBduk (https://sourceforge.net/projects/bbmap/) to remove adapters and low-quality sequences, resulting in an average of 22.89 million surviving reads per library. Filtered reads were mapped to the wild-type strain assembly with HISAT2 v2.1 [159] with average mapping rates of 98.5% per sample.

HTSeq-count v0.11.1 [160] was used to generate counts at annotated genes, which were passed to DESeq2 v1.30.1 [161,162] for further analysis. We removed from analysis a single library from an evolved isolate grown in maltose, as manual inspection of normalized gene expression values revealed that this sample had stochastically lost the ChrXV aneuploidy. This reduced our power to detect statistically significant differences in expression for that specific evolved isolate. All other samples from evolved isolates remained aneuploid in both conditions. We considered DEGs between conditions and genotypes with expression changes of greater than or equal to 2-fold in either direction and Benjamini–Hochberg adjusted *p*-values of less than or equal to 0.01 (false discovery rate of 1%). Full differential expression analysis results can be found in S4 Table. To compare expression levels of single genes, we used size-normalized counts from DESeq2, which are more robust for this purpose than other normalization methods [163–165]. We defined subtelomeric genes as those falling within 20 kb of the end of a contig, which represented entire chromosomes in our assembly (with the exception of the two-contig ChrXII, for which we considered genes within 20 kb of the telomeric contig ends, not the ends containing *rDNA* repeats). This classification is comparable to or more conservative than those used previously [108,166]. GOrilla [167] was used to identify enriched gene ontology (GO) terms in gene sets of interest; we used *S*. *cerevisiae* GO annotations and specified all predicted genes in our annotation as the background set against which to test for enrichment. Statistics and data visualization were performed in R.

### RT-qPCR

For the experiment shown in S9 Fig, 100 ng total RNA was used as input for the Luna Universal One-Step RT-qPCR kit (New England Biolabs), with cycling and data acquisition performed on an Applied Biosystems 7500 Real-Time System (Thermo Fisher Scientific). Relative expression of *AGT1* was analyzed using the ΔΔC_T_ method with normalization to *ACT1* and *ARP2* [168].

### Ploidy determination

Flow cytometry-based ploidy determination was performed as described previously [7], except that we sampled asynchronous cultures. Briefly, we fixed mid-log cultures of each query, treated fixed cells with RNAse A and Proteinase K, and stained DNA with Sytox Green (Thermo Fisher Scientific). Haploid and diploid *S*. *cerevisiae* strains were included in all experiments as controls. For clonal strains (*S*. *cerevisiae* controls, ancestral *S*. *eubayanus*, and evolved isolates), queries were streaked to single colonies, and independent colonies were picked for ploidy analysis. For population samples, entire populations cryopreserved at −80°C in 15% glycerol were gently thawed, and approximately 50 μL was inoculated directly to 1 mL SC-2% maltose. Cells were harvested and fixed in early log phase after a minimum of 2 doublings, and 10,000 cells were sampled for each query on an Attune NxT flow cytometer (Thermo Fisher Scientific). Analysis was performed in FlowJo v10.

### Fitness assays

Except for experiments in rich medium shown in Fig 2A, the conditions for fitness assays were designed to mimic the original ALE conditions [64]. Briefly, this regime consisted of culturing in 1 mL SC medium with 2% maltose and 0.1% glucose (hereafter, “competition medium”) with semiweekly 1:10 dilutions into new competition medium. Query genotypes were directly competed against a common competitor in co-culture. The competitor was a haploid in the ancestral *S*. *eubayanus* strain background with the exception of a constitutively expressed GFP using a *TEF1* promoter and *ADH1* terminator from *S*. *cerevisiae* and *ste12* deletion (*MAT***a** *hoΔ*::*P*_*ScTEF1*_*-yEGFP-T*_*ScADH1*_*-kanMX* *ste12Δ*::*natMX*). We chose a *ste12* deletion to prevent any interaction with competitors expressing *MAT***α.** Strains were streaked to single colonies on YPD containing antibiotic as appropriate, precultured in competition medium for 3 days, mixed in approximately equal query-to-competitor ratios (except where we reduced the competitor ratio against less-fit query genotypes), sampled into cold 1× PBST for flow cytometry of time point 0, and inoculated into 1 mL competition medium at an initial OD_600_ of approximately 0.1. At each transfer, competitions were sampled into cold 1× PBST for flow cytometry, and the optical density of each replicate was measured to calculate the number of generations. Competitions in rich medium were carried out in the same manner, albeit that preculturing and propagation were in sterile-filtered YPD in 2 mL volume with daily dilutions of 1:100. For both competition regimes, we sampled 13,000 cells per replicate and time point on an Attune NxT flow cytometer (Thermo Fisher Scientific) to quantify the abundance of competitor (fluorescent) and query (non-fluorescent) cells, which always clearly formed distinct populations. Analysis was performed in FlowJo v10. Fitness was calculated as the selection coefficient, obtained by regressing the natural log ratio of query to competitor against the number of generations. To analyze the effects of ploidy, mating type, and cell type (diploid-like and haploid-like) on the panel of strains shown in Fig 3, we used multiple linear regression with measured fitness as the response and ploidy, mating type, and cell type as categorical predictors with 2 levels each (for mating type, we grouped by whether genotypes expressed any mating type-specific genes, or none). All statistical analyses and visualization were performed in R.

### *P*_*AGT1*_ reporter analysis

We generated single-copy genome integrations in haploids of yeast-enhanced GFP (*yEGFP*) expressed from both the native *AGT1* promoter and a variant in which we abolished the Tec1p consensus site (TCS) by making point mutations to each of its 6 nucleotides (*P*_*agt1-tcs*_). To compare expression between *P*_*AGT1*_ and *P*_*agt1-tcs*_, strains were streaked to single colonies on YPD plates, picked to SC-2% maltose and grown to saturation, back-diluted in 2 mL SC-2% maltose to an initial OD_600_ of 0.01, and grown to mid-log phase. Cells were collected by centrifugation, washed twice with cold PBST, and resuspended in PBST for flow cytometry. We sampled 40,000 cells per replicate on an Attune NxT (Thermo Fisher Scientific). Analysis was performed in FlowJo v10, and fluorescence values were exported for statistical analysis and visualization in R. A similar approach was taken to test the carbon source-dependence of *P*_*AGT1*_*-GFP*, albeit that precultures and cultures were inoculated into SC-2% glucose, SC-2% maltose, and SC-2% methyl-*α*-glucoside and grown to mid log phase. To test reporter expression in the no-carbon condition, cultures pre-grown in glucose were inoculated into SC medium at an initial OD_600_ of approximately 0.3 and incubated for the same duration as the maltose cultures. Bulk fluorescence was measured on a BMC FLUOstar Omega plate reader at a cell density of OD_600_ = 1 and background-normalized.

## Supporting information

S1 Fig Details of aneuploidy in evolved clones. Dot plot of copy number variation in the evolved isolates. Relative copy number, inferred from sequencing depth, is plotted for each chromosome with a small amount of x-axis jitter. Relative coverage of *AGT1* on ChrXV is indicated by filled dots, indicating the absence of CNVs beyond aneuploidy. The data underlying this figure can be found in S1 Data.
(TIF)

S2 Fig Evolved clones possess only a single allele at each variable site. Genome browser tracks showing aligned Illumina reads from the population 1 clone at *SIR4* (a), *IRA1* (b), and *YDJ1* (c), and from the population 2 clone at *LAM5* (d). Mapped reads are depicted as gray bars with mismatches colored according to base identity. The data underlying this figure can be found in S1 Data.
(TIF)

S3 Fig Ploidy variation across the adaptive evolution experiment. (a) Smoothed histograms of cellular DNA content for asynchronous haploid (top panel) and diploid (middle panel) *S*. *cerevisiae* (*Sc*) controls and the wild-type *S*. *eubayanus* (*Se* Anc.) strain (bottom panel, reproduced from the same data as in Fig 1). (b) Histograms for population-level samples from both ALE replicates (gray) and clonal isolates from each population (red shades). For clones, the 2 histograms represent results from independent experiments; the bottom panel for each (dark red) is the same data displayed in Fig 1. For population samples, panels are arranged from top to bottom with increasing time and number of ALE generations, representing approximately 50 generation intervals from 50–350. The bottom panel for each population represents the terminal time point from which the adapted clones were isolated and from which we quantitatively assessed haploid frequency. (c) Fraction of haploids in the terminal time point of each ALE population assayed by *MAT* locus PCR genotyping. Points and bars show the mean and standard error of 4 experiments. The data underlying this figure can be found in S1 Data.
(TIF)

S4 Fig Temporal dynamics of ploidy variants in evolving populations. (a) Aneuploidy frequency across the adaptive evolution experiment. The relative copy number of each chromosome, inferred from sequencing depth, is plotted for whole-population samples from the ALE experiment from approximately 50 generation intervals. The trajectories of aneuploidies that reached high frequencies are colored; all other chromosomes are black. The time points are the same as those sampled to assay ploidy states (S3B Fig). (b) Aneuploidies in whole-population samples are plotted against generations as in (a), but they are rescaled to frequency per haploid genome. The apparent frequency of haploids in each population from the same time points is plotted as green lines and was calculated from the flow cytometry data shown in S3B Fig. The data underlying this figure can be found in S1 Data.
(TIF)

S5 Fig Increased copy number of *AGT1* is adaptive. (a) Boxplots show the differences in fitness of diploids and haploids with an extra copy of *AGT1*, compared to the respective parent strain. While haploids experience a smaller change in fitness than diploids, the overall fitness of haploids with increased *AGT1* expression is significantly and substantially higher than that of diploids with increased *AGT1* expression (b). The data underlying this figure can be found in S1 Data.
(TIF)

S6 Fig Transcriptomes of the independently evolved haploids are similar. (a) Principal component (PC) plot of normalized gene expression for the mRNA-seq libraries used here. Points represent individual libraries, colored by strain and growth condition (evol1, evol2: evolved haploids; ancestor: wild-type diploid). (b) Scatterplot of the relative expression of all genes in both conditions for each evolved haploid with hexbin color indicating the density of points. Pearson’s *ρ* is given inset (*p* < 2.2 × 10^−16^). The data underlying this figure can be found in S1 Data.
(TIF)

S7 Fig Additional gene expression comparisons. (a) Boxplots show log_2_-transformed fold changes (LFC) of gene expression on glucose (instead of maltose, as in Fig 4) in evolved haploids compared to the wild-type strain for genes on aneuploid ChrXV (*n* = 370) and subtelomeric genes (*n* = 200). (b) Boxplots show LFCs of gene expression in maltose compared to glucose for ChrXV genes in each evolved haploid. Whiskers extend to 1.5× the interquartile range. Lines connect the y-axis coordinates of the same gene in each evolved isolate; axes are scaled such that an occasional outlier is truncated from the plot space for a single strain. *AGT1* expression is plotted as red dots and lines, and black dashed lines indicate the null expectation for expression values between strains (a) or equivalent expression between conditions (b). The data underlying this figure can be found in S1 Data.
(TIF)

S8 Fig *P*_*AGT1*_*-GFP* reporter expression in 5 carbon conditions. Boxplots show normalized fluorescence measurements of *P*_*AGT1*_*-GFP*-expressing strains in 5 SC media conditions: glucose (GLUC), galactose (GAL), methyl-*α*-glucoside (MAG), maltose (MAL), and no carbon (NC) with *n* = 9 biological replicates each. Conditions that differ significantly from glucose are indicated (****p = 4.1 × 10^−5^, Mann–Whitney *U* tests). The data underlying this figure can be found in S1 Data.
(TIF)

S9 Fig RT-qPCR of *AGT1* in euploids. Bars show mean and standard deviation of *AGT1* expression in euploid diploids and haploids grown in SC-2% maltose as measured by RT-qPCR. The growth conditions in this preliminary experiment seem not to have matched those in the RNA-seq experiments. The difference in haploid-specific *AGT1* expression between these experiments likely reflects this large batch effect or it could suggest an additional interaction with aneuploidy as discussed in the main text. The data underlying this figure can be found in S1 Data.
(TIF)

S1 Table Strains and plasmids used in this study. (XLSX)

S2 Table Oligonucleotides used in this study. (XLSX)

S3 Table Mutations in evolved isolates. (XLSX)

S4 Table Full differential expression analysis results. (XLSX)

S5 Table All putative transcription factor motifs identified in the *AGT1* promoter. (TXT)

S1 Data Data underlying all figures. (XLSX)

## Abbreviations

ALE: adaptive laboratory evolution
CNV: copy number variant
DEG: differentially expressed gene
GO: gene ontology
SNP: single-nucleotide polymorphism
TCS: TEA/ATTS consensus sequence
