# Supplementary figures and images for: Ploidy evolution in a wild yeast is linked to an interaction between cell type and metabolism
Source: PLoS Biol. 2023 Nov 9;21(11):e3001909. doi: 10.1371/journal.pbio.3001909 (PMC10635434; doi:10.1371/journal.pbio.3001909)

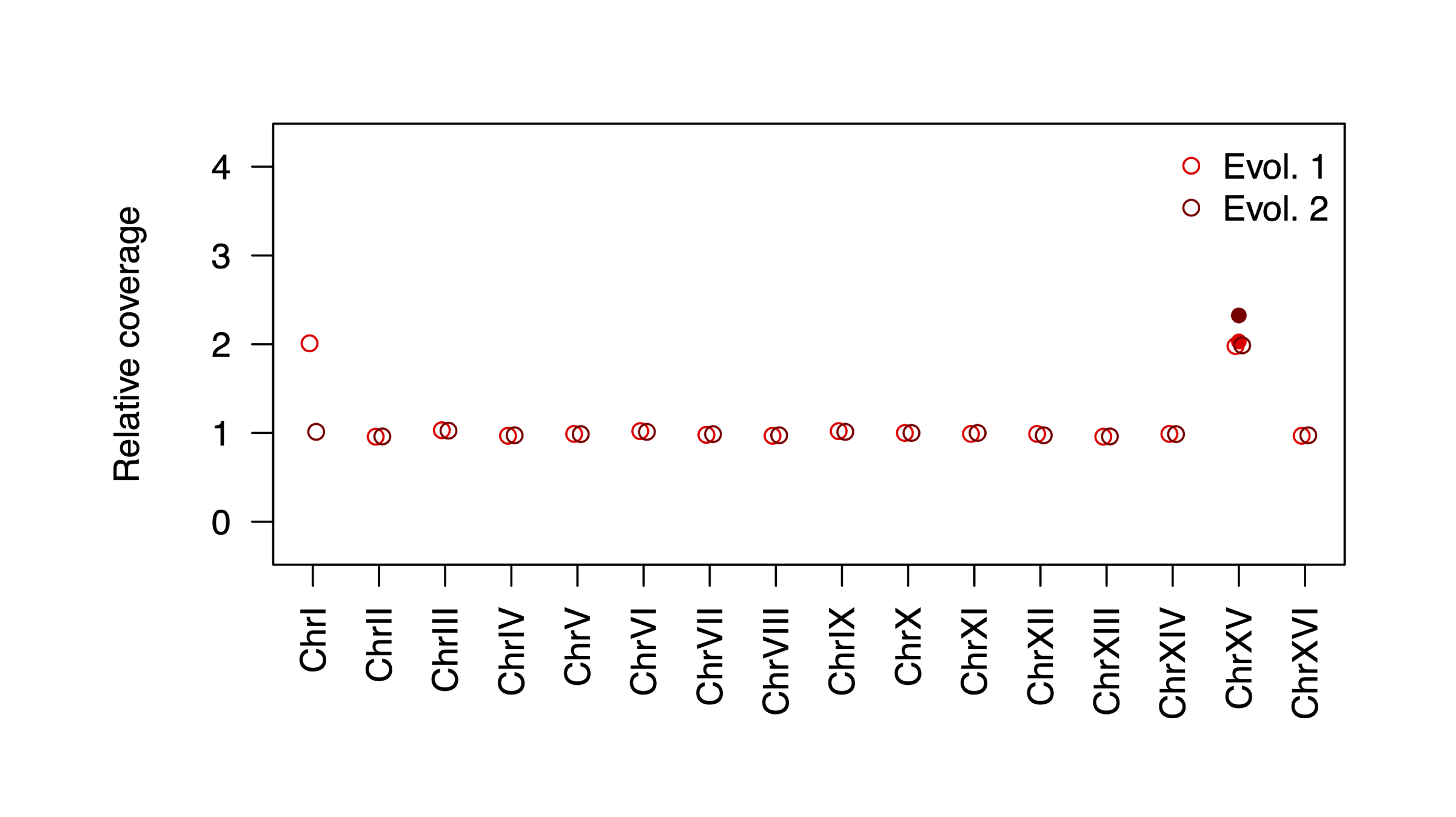

Supplement: S1 Fig — Dot plot of copy number variation in the evolved isolates. Relative copy number, inferred from sequencing depth, is plotted for each chromosome with a small amount of x-axis jitter. Relative coverage of AGT1 on ChrXV is indicated by filled dots, indicating the absence of CNVs beyond aneuploidy. The data underlying this figure can be found in S1 Data. (TIF) [file pbio.3001909.s001.tif]

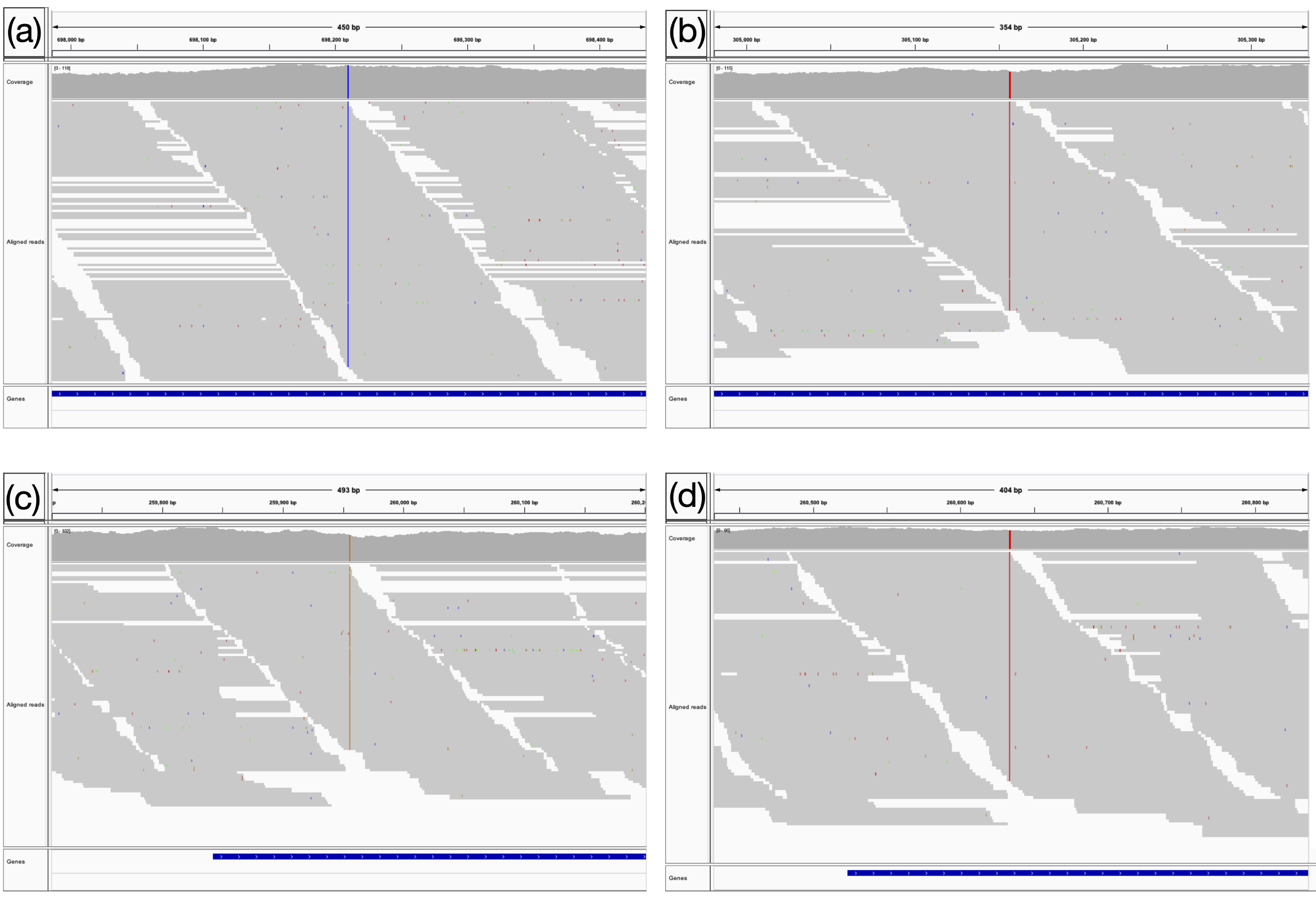

Supplement: S2 Fig — Genome browser tracks showing aligned Illumina reads from the population 1 clone at SIR4 (a), IRA1 (b), and YDJ1 (c), and from the population 2 clone at LAM5 (d). Mapped reads are depicted as gray bars with mismatches colored according to base identity. The data underlying this figure can be found in S1 Data. (TIF) [file pbio.3001909.s002.tif]

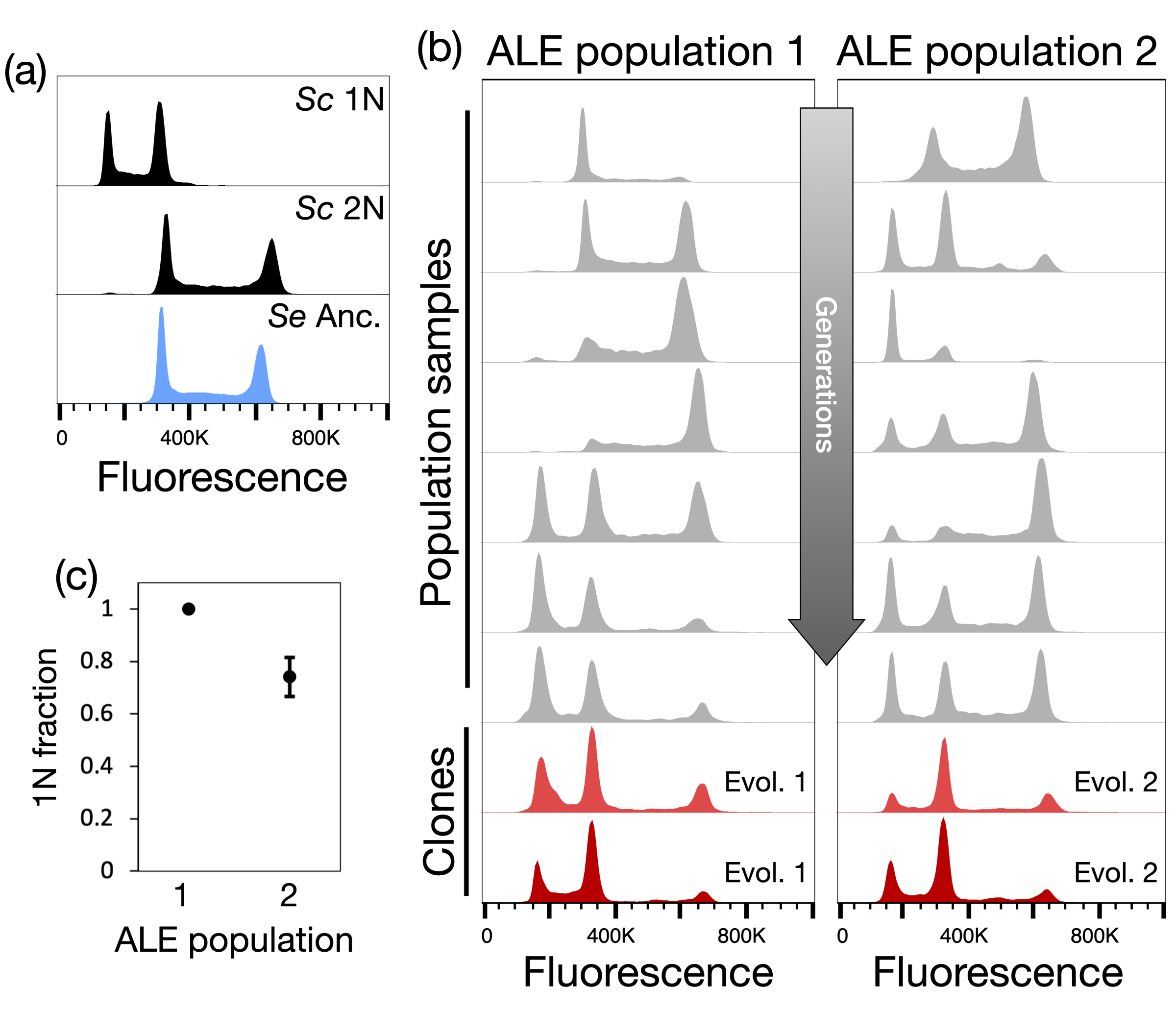

Supplement: S3 Fig — (a) Smoothed histograms of cellular DNA content for asynchronous haploid (top panel) and diploid (middle panel) S. cerevisiae (Sc) controls and the wild-type S. eubayanus (Se Anc.) strain (bottom panel, reproduced from the same data as in Fig 1). (b) Histograms for population-level samples from both ALE replicates (gray) and clonal isolates from each population (red shades). For clones, the 2 histograms represent results from independent experiments; the bottom panel for each (dark red) is the same data displayed in Fig 1. For population samples, panels are arranged from top to bottom with increasing time and number of ALE generations, representing approximately 50 generation intervals from 50–350. The bottom panel for each population represents the terminal time point from which the adapted clones were isolated and from which we quantitatively assessed haploid frequency. (c) Fraction of haploids in the terminal time point of each ALE population assayed by MAT locus PCR genotyping. Points and bars show the mean and standard error of 4 experiments. The data underlying this figure can be found in S1 Data. (TIF) [file pbio.3001909.s003.tif]

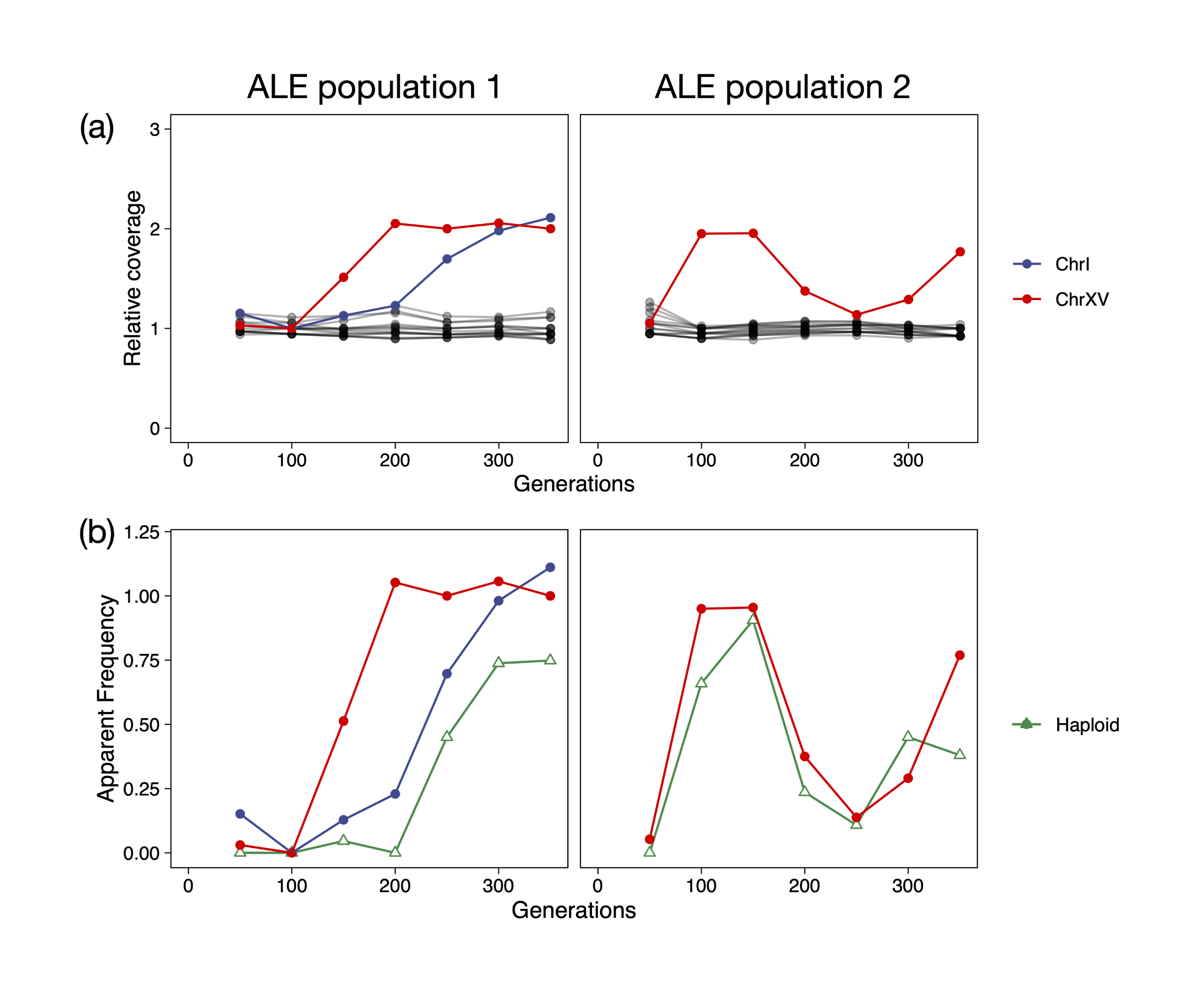

Supplement: S4 Fig — (a) Aneuploidy frequency across the adaptive evolution experiment. The relative copy number of each chromosome, inferred from sequencing depth, is plotted for whole-population samples from the ALE experiment from approximately 50 generation intervals. The trajectories of aneuploidies that reached high frequencies are colored; all other chromosomes are black. The time points are the same as those sampled to assay ploidy states (S3B Fig). (b) Aneuploidies in whole-population samples are plotted against generations as in (a), but they are rescaled to frequency per haploid genome. The apparent frequency of haploids in each population from the same time points is plotted as green lines and was calculated from the flow cytometry data shown in S3B Fig. The data underlying this figure can be found in S1 Data. (TIF) [file pbio.3001909.s004.tif]

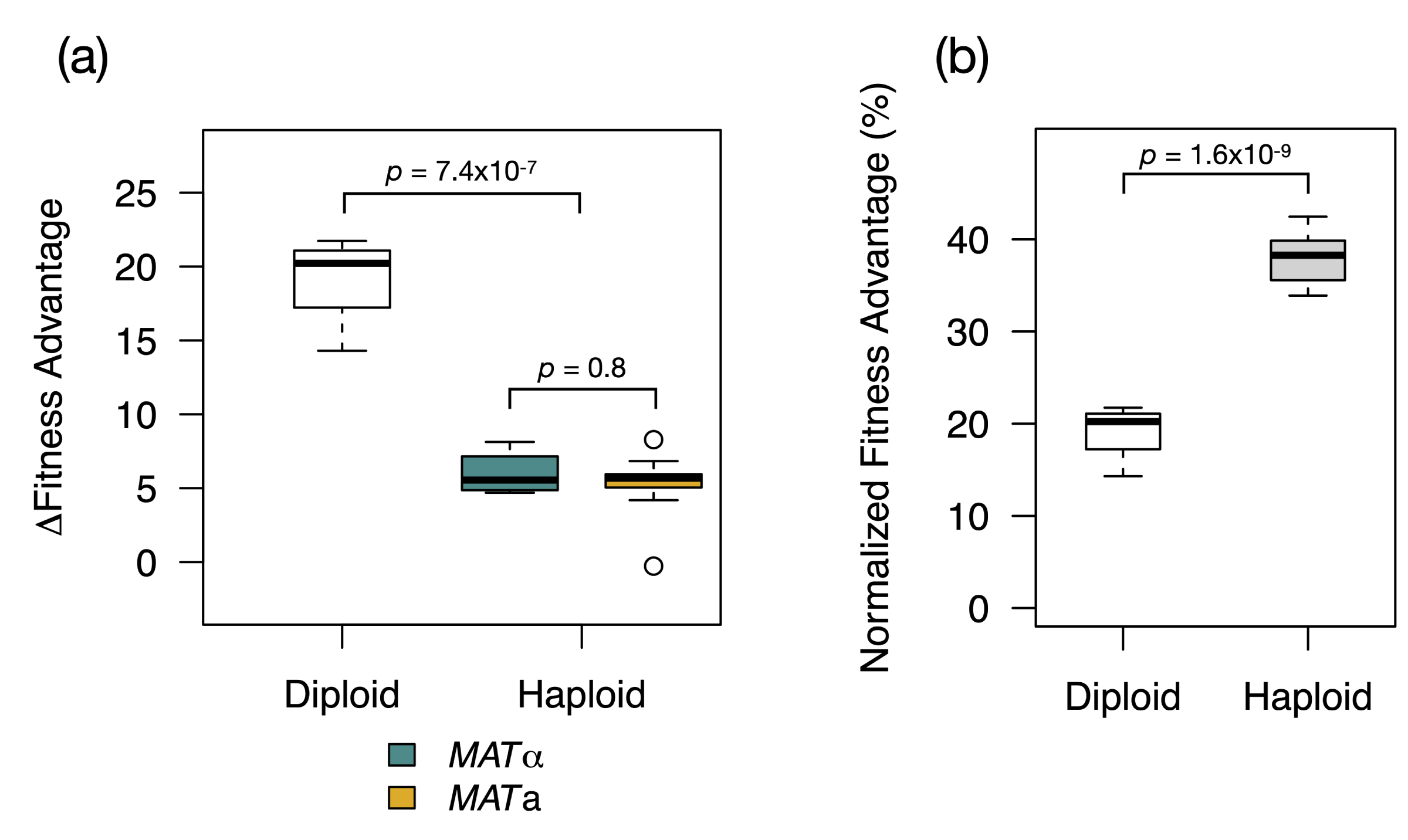

Supplement: S5 Fig — (a) Boxplots show the differences in fitness of diploids and haploids with an extra copy of AGT1, compared to the respective parent strain. While haploids experience a smaller change in fitness than diploids, the overall fitness of haploids with increased AGT1 expression is significantly and substantially higher than that of diploids with increased AGT1 expression (b). The data underlying this figure can be found in S1 Data. (TIF) [file pbio.3001909.s005.tif]

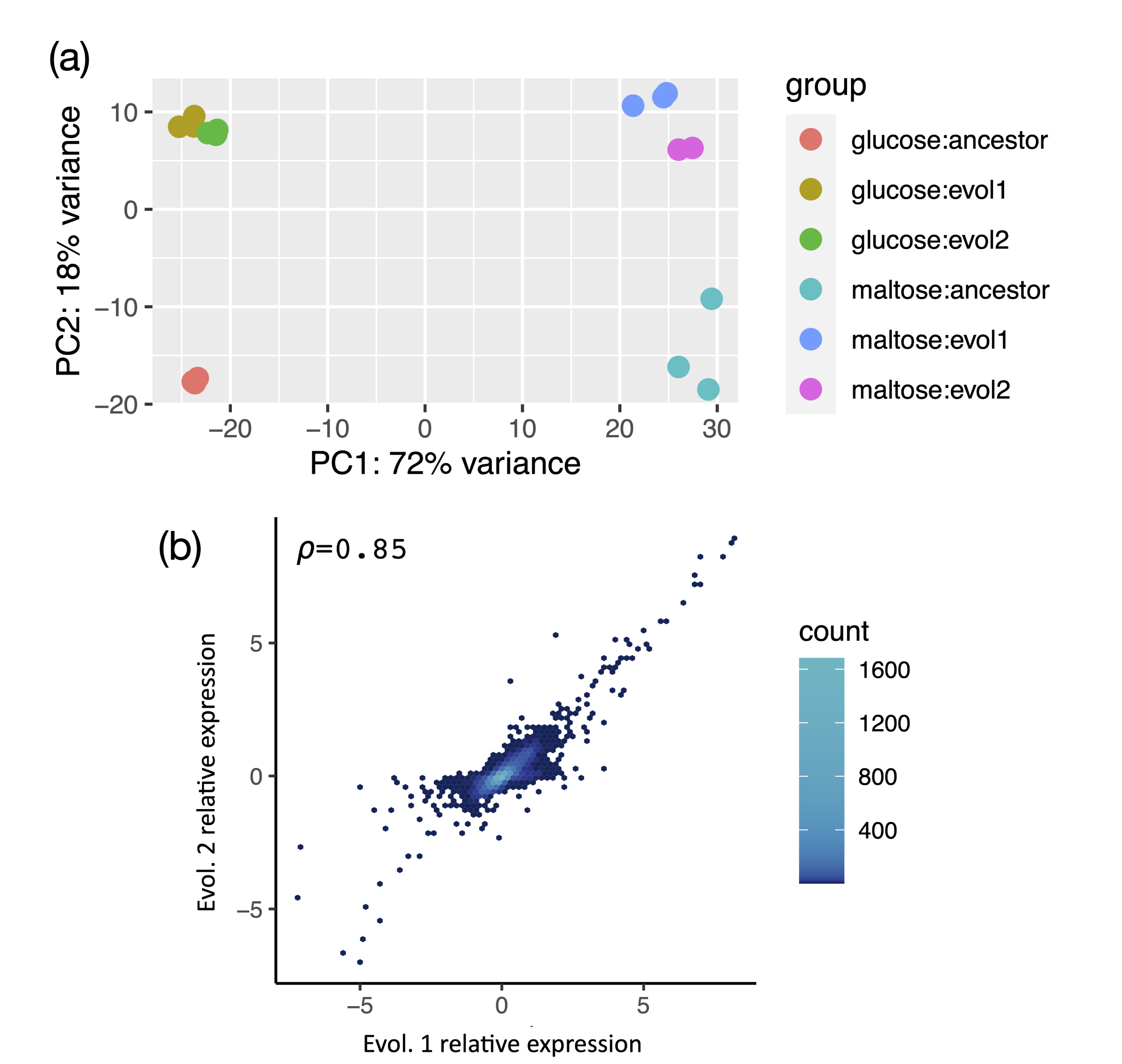

Supplement: S6 Fig — (a) Principal component (PC) plot of normalized gene expression for the mRNA-seq libraries used here. Points represent individual libraries, colored by strain and growth condition (evol1, evol2: evolved haploids; ancestor: wild-type diploid). (b) Scatterplot of the relative expression of all genes in both conditions for each evolved haploid with hexbin color indicating the density of points. Pearson’s ρ is given inset (p < 2.2 × 10^−16^). The data underlying this figure can be found in S1 Data. (TIF) [file pbio.3001909.s006.tif]

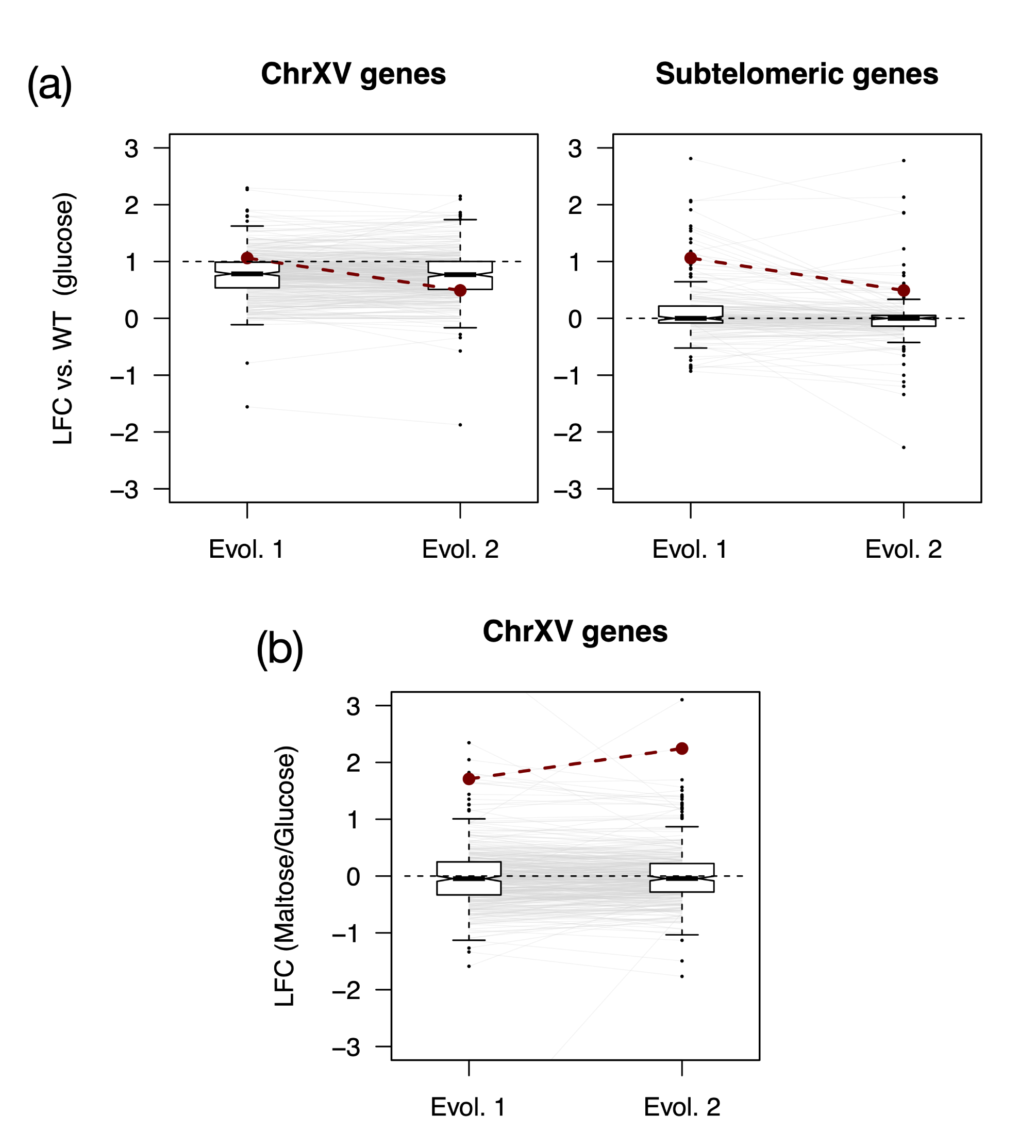

Supplement: S7 Fig — (a) Boxplots show log2-transformed fold changes (LFC) of gene expression on glucose (instead of maltose, as in Fig 4) in evolved haploids compared to the wild-type strain for genes on aneuploid ChrXV (n = 370) and subtelomeric genes (n = 200). (b) Boxplots show LFCs of gene expression in maltose compared to glucose for ChrXV genes in each evolved haploid. Whiskers extend to 1.5× the interquartile range. Lines connect the y-axis coordinates of the same gene in each evolved isolate; axes are scaled such that an occasional outlier is truncated from the plot space for a single strain. AGT1 expression is plotted as red dots and lines, and black dashed lines indicate the null expectation for expression values between strains (a) or equivalent expression between conditions (b). The data underlying this figure can be found in S1 Data. (TIF) [file pbio.3001909.s007.tif]

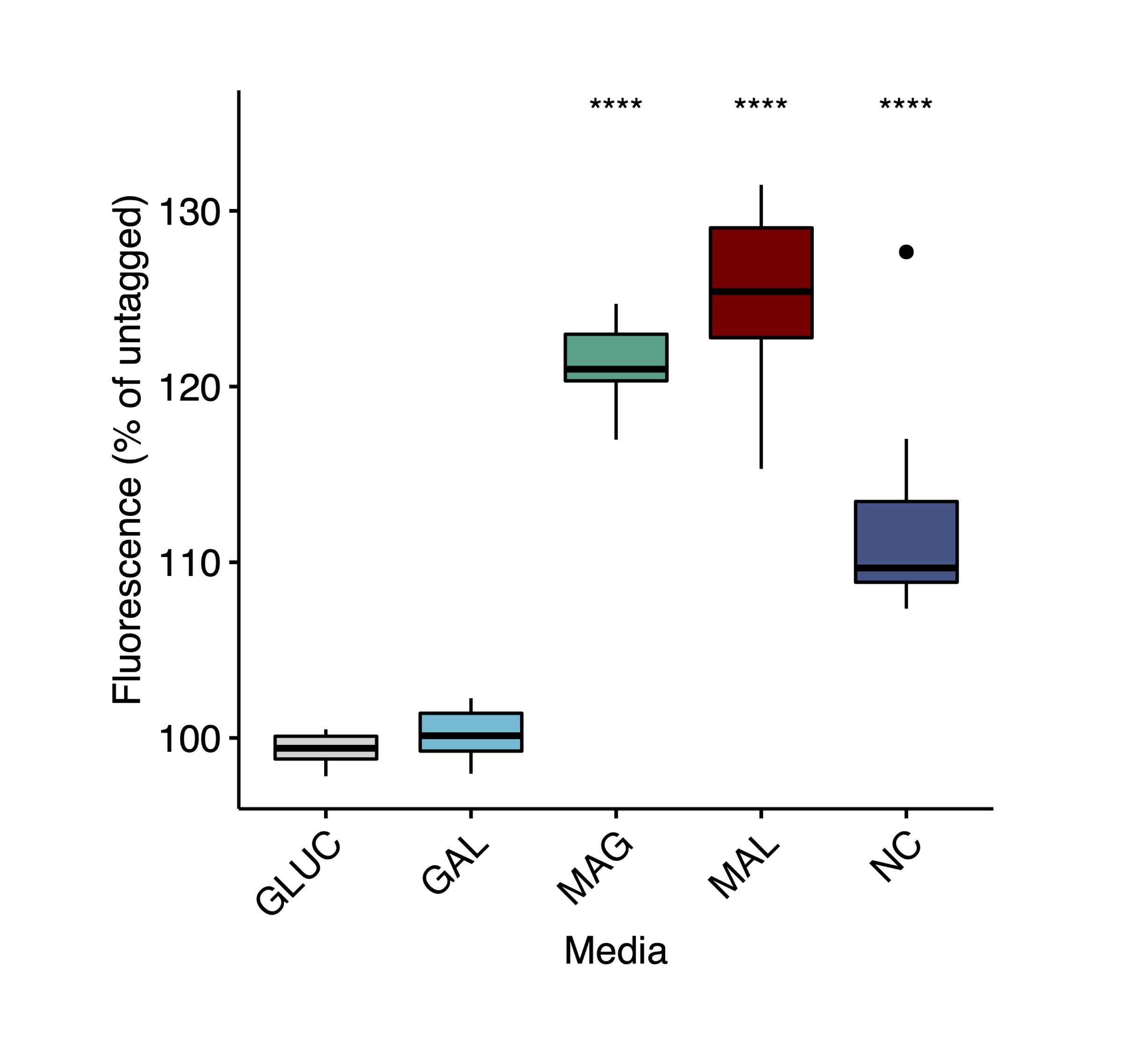

Supplement: S8 Fig — Boxplots show normalized fluorescence measurements of PAGT1-GFP-expressing strains in 5 SC media conditions: glucose (GLUC), galactose (GAL), methyl-α-glucoside (MAG), maltose (MAL), and no carbon (NC) with n = 9 biological replicates each. Conditions that differ significantly from glucose are indicated (****p = 4.1 × 10^−5^, Mann–Whitney U tests). The data underlying this figure can be found in S1 Data. (TIF) [file pbio.3001909.s008.tif]

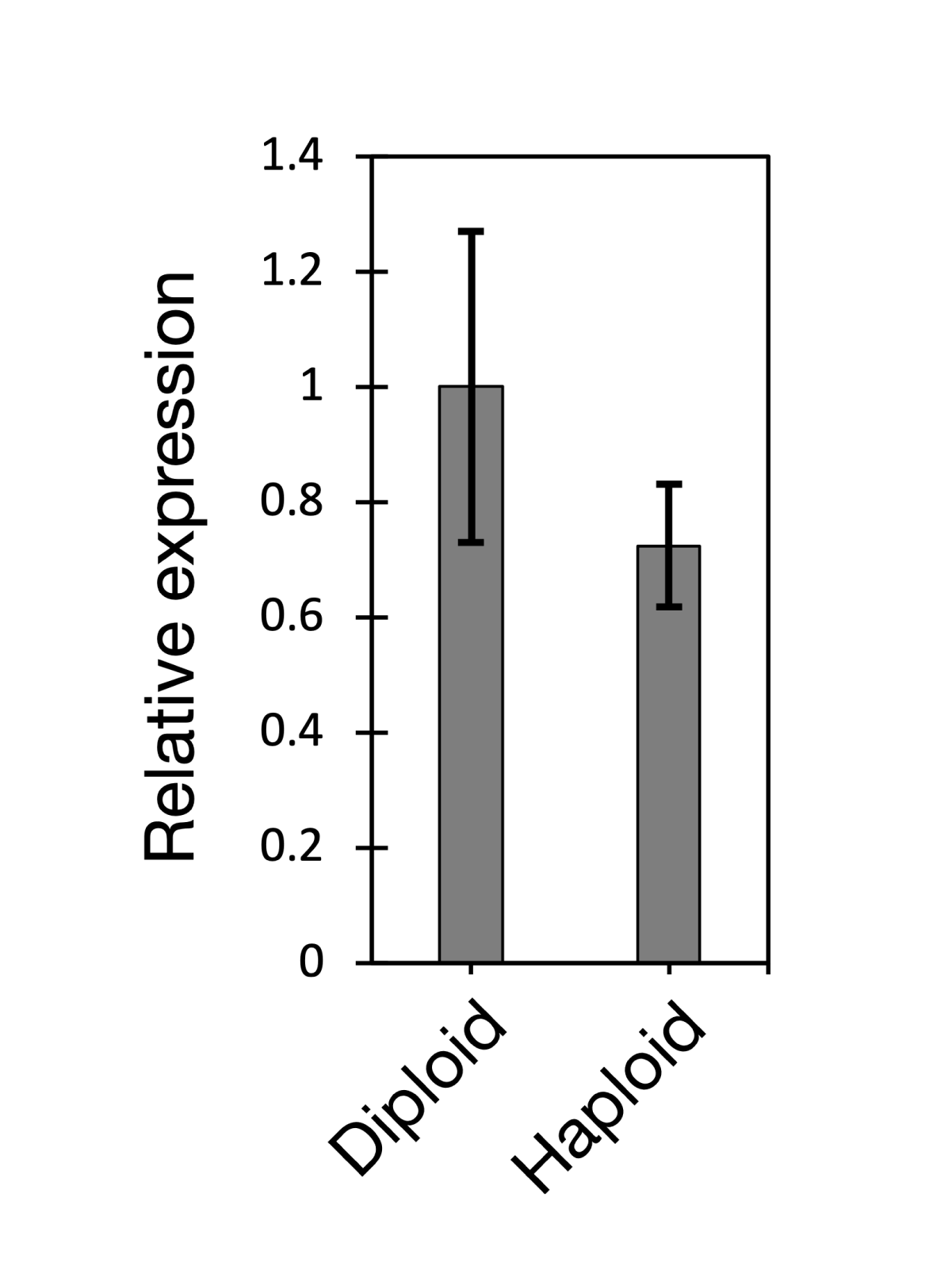

Supplement: S9 Fig — Bars show mean and standard deviation of AGT1 expression in euploid diploids and haploids grown in SC-2% maltose as measured by RT-qPCR. The growth conditions in this preliminary experiment seem not to have matched those in the RNA-seq experiments. The difference in haploid-specific AGT1 expression between these experiments likely reflects this large batch effect or it could suggest an additional interaction with aneuploidy as discussed in the main text. The data underlying this figure can be found in S1 Data. (TIF) [file pbio.3001909.s009.tif]
